# Integrative blood profiling uncovers inflammatory network signatures in high-altitude pulmonary edema

**DOI:** 10.1042/BSR20253746

**Published:** 2025-12-12

**Authors:** Kanika Singh, Krishna Kumar G, Manzoor Ali, Raushni Choudhary, Mohit Khadia, Stanzen Rabyang, Tashi Thinlas, Rahul Kumar, Aastha Mishra

**Affiliations:** 1 Genomics and Genome Biology Unit, CSIR-Institute of Genomics and Integrative Biology, Delhi, 110025, India; 2 Academy of Scientific and Innovative Research (AcSIR), Ghaziabad, 201002, India; 3 Department of Medicine, Sonam Norboo Memorial Hospital, Leh, 194101, India; 4 Department of Medicine, University of California San Francisco, San Francisco, California, 94143, U.S.A.; 5 Lung Biology Center, Zuckerberg San Francisco General Hospital, San Francisco, California, 94143, U.S.A.

**Keywords:** high-altitude pulmonary edema, hypobaric hypoxia, lncRNA profiling, inflammatory signature, multiomics approach, transcriptomics

## Abstract

Despite the well-known role of hypoxia-driven inflammatory mediators in the pathogenesis of hypoxic pulmonary hypertension, their involvement in high-altitude (HA) illnesses, particularly high-altitude pulmonary edema (HAPE), remains unclear. The present study uses an integrated clinical, transcriptomic, proteomic, and long noncoding RNA (lncRNA) profiling of 83 individuals, including HAPE patients segregated into mild, moderate, and severe categories, HAPE-free sojourners, and long-term HA residents, to highlight the molecular and immunological changes associated with HAPE and its severity. Clinical assessments revealed significantly reduced peripheral oxygen saturation and elevated respiratory parameters in HAPE patients. Differential gene expression and functional enrichment analyses identified 515 significantly differentially expressed genes, with marked enrichment of inflammatory and hypoxia-associated pathways. Protein–protein interaction network analysis revealed eighteen hub genes, including toll-like receptor (TLR) 2 and Forkhead box O3 (FOXO3), with strong diagnostic potential. Immune cell deconvolution analysis and hematological profiling indicated a prominent increase in neutrophil proportion. Interestingly, oncostatin M (OSM), a hypoxia-regulated predominant cytokine produced by neutrophils, was revealed in the cytokine and transcriptomic profiling, highlighting its role in inflammation and extracellular matrix degradation. Co-expression network analysis notably revealed significant alterations that formed a gene module exhibiting a strong correlation with immune response, leukocyte adhesion, and ncRNA processing pathways. Interestingly, these co-expressed partners, LINC01093 and immune-regulatory genes like interleukin-18 receptor 1 and TLR5, appear to regulate the NF-κB signaling pathway, one of the positively enriched pathways in our analysis. Overall, this multiomics approach highlighted a strong inflammatory signature and lncRNA-mRNA interactions associated with HAPE.

## Introduction

High altitude (HA) presents a physiologically challenging environment due to reduced oxygen availability, triggering various cellular and metabolic adaptations to maintain homeostasis [1]. Globally, approximately 81.6 million individuals reside at elevations above 2,500 m [2], while over 100 million lowlanders visit HA annually for recreational or occupational purposes, including tourists, trekkers, miners, construction workers, and military personnel [2,3]. However, a subset of individuals develops altitude-associated illnesses, influenced by genetic and epigenetic susceptibility or pre-existing health conditions. A critical yet understudied factor in HA illnesses is the interplay between hypoxia and inflammation [4–6]. While hypoxia-driven inflammatory mediators play a well-documented role in the pathogenesis of hypoxic pulmonary hypertension (PH) [7–9], their involvement in other HA illnesses, particularly high-altitude pulmonary edema (HAPE), remains unclear. HAPE is a severe, potentially fatal noncardiogenic pulmonary edema that occurs in otherwise healthy but susceptible individuals upon rapid ascent to HA [10,11]. It manifests clinically with dyspnea, tachypnea, tachycardia, persistent cough, fatigue, cyanosis, pink frothy sputum, and characteristic chest X-ray findings of increased lung vascular markings and patchy infiltrates [12]. Pathologically, HAPE results from an imbalance in intrapulmonary fluid dynamics, leading to excessive capillary leakage into the alveoli, interstitium, and bronchioles, ultimately impairing gas exchange. Despite the well-known phenomenon that hypoxia triggers an early inflammatory response [8,9,13], the role of inflammatory immune cells and the associated inflammatory molecular pathways in driving these abnormal vascular pathologies in HAPE remains controversial [14].

Of note, several findings suggest an inflammatory component, including the efficacy of dexamethasone, a glucocorticoid with anti-inflammatory properties, in treating HAPE patients [15,16]. The presence of upper respiratory tract infections has been associated with increased susceptibility to HAPE [17], while elevated urinary leukotriene metabolites and pro-inflammatory cytokines in bronchoalveolar lavage samples further support the involvement of inflammatory mediators [11,18,19]. Our recent findings show significantly higher levels of High mobility group Box 1 protein, which is known to elicit inflammatory pathways in HAPE [20]. Recruitment of inflammatory immune cells in acute hypoxic PH, a hallmark of HAPE, and up-regulation of key plasma biomarkers of the vascular homeostasis and oxidative stress pathway in HAPE patients highlight the potential contribution of inflammation [21,22]. However, earlier studies have failed to detect inflammatory biomarkers in the circulation, bronchoalveolar lavage, or urine of HAPE patients, leading to skepticism regarding its role [23–25]. This discrepancy may stem from inadequate biomarker selection or the unavailability of sensitive analytical tools capable of detecting subtle inflammatory cascades.

With recent advancements in multiomics technologies, there is now an opportunity to explore the inflammatory landscape of HAPE in greater depth. We hypothesize that a deeper phenotyping of circulatory biomarkers, along with novel approaches such as long noncoding RNA (lncRNA) profiling, could identify predisposing or prognostic biomarkers and clarify the role of inflammation in HAPE pathogenesis. To address these questions, we conducted a comprehensive high-throughput blood profiling study in HAPE patients, acclimatized HAPE-free sojourners (Controls), and long-term HA residents (HLs). Our approach integrated whole transcriptome analysis using RNA sequencing with targeted proteomic profiling of plasma samples. The findings revealed significant enrichment of hallmark inflammatory pathways and altered immune cell composition in HAPE patients, with targeted plasma cytokine profiles corroborating transcriptomic insights. Additionally, weighted gene co-expression network analysis (WGCNA) identified interactions between coding and long ncRNAs, offering new perspectives on the molecular mechanisms underlying HAPE. By leveraging multiomics data and specialized computational tools, our study provides a comprehensive understanding of gene expression alterations under hypobaric hypoxia and highlights the potential role of inflammation in HAPE pathophysiology.

## Methods and materials

### Study approval

The human ethical committees of the CSIR-Institute of Genomics and Integrative Biology (IGIB), Delhi, and Sonam Norboo Memorial Hospital (SNMH), Leh provided approval for the study. The study was conducted in compliance with the principles of the Declaration of Helsinki. The identities of all participants were kept confidential, and their blood samples were taken only after the submission of written informed consent.

### Study participant selection

**Figure 1:**
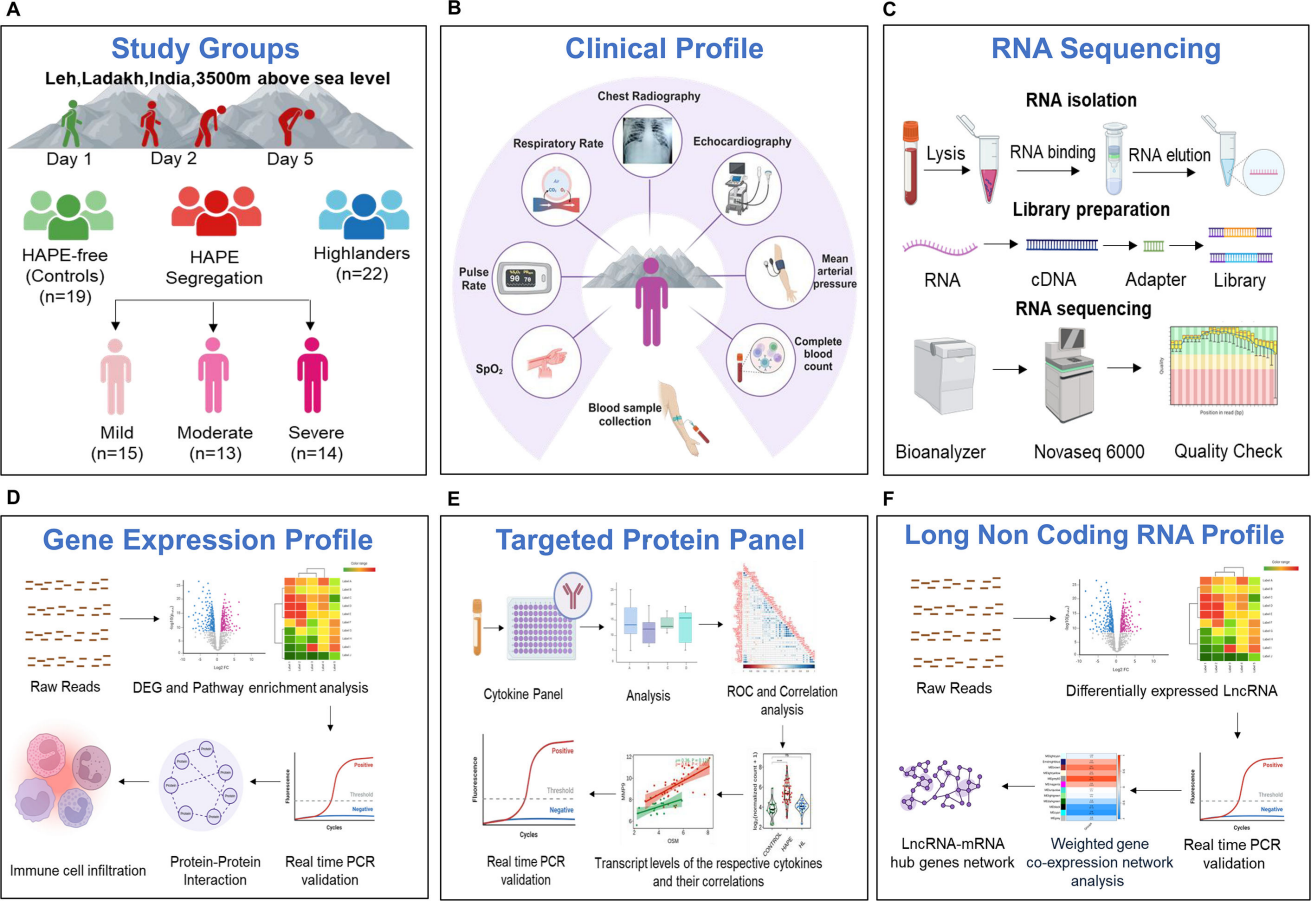
Schema of the study design. (**A**) The three study groups, HAPE-free sojourners, HAPE patients, and highlanders. (**B**) Clinical parameters recorded for the study participants. (**C**) Steps involved in RNA sequencing include RNA isolation from blood, library preparation, and sequencing. (**D**) Gene expression profile and immune cell abundance analysis. (**E**) Targeted protein panel analysis and transcript levels of the corresponding cytokines. (**F**) The Differentially expressed lncRNA profile and WGCNA analysis for lncRNA and mRNA co-expressed genes.

As shown in Figure 1A, the study included 95 participants aged between 18 and 70 years. They were classified into three distinct study groups: healthy HA sojourners or controls (*n* = 25); HAPE patients segregated into mild (*n* = 15), moderate (*n* = 15), and severe categories (*n* = 15); and healthy HLs (*n* = 25). The segregation of HAPE into three categories was based on the clinical signs, symptoms, and chest X-ray images as described previously [10,20]. HAPE patients included sojourners from low-altitude regions (<200 m) who traveled to Leh, Ladakh, and developed HAPE within two to five days of HA exposure. Healthy HA sojourners or controls included sojourners from low-altitude regions (<200 m) who visited HA under similar conditions as the HAPE group but did not develop any HA-related disorders. Lake Louise scoring was applied to rule out acute mountain sickness in controls [26]. The HL group comprises healthy individuals who are long-term residents of Leh, Ladakh, having an altitude of 3500 m above sea level (asl).

### Study criteria and biospecimen collection

Exclusion criteria included patients with comorbid conditions, including hematological or cardiovascular diseases such as diabetes, hypertension, valvular disease, or other cardiac anomalies, patients who had received HAPE treatment, including supplemental oxygen, before blood sample collection, and patients with other causes of hypoxemia, such as pneumonia, or concurrent HA illnesses, including high-altitude cerebral edema, as determined through clinical assessment. A 10 ml of blood samples from each participant of the study groups was collected immediately after diagnosis but before the start of the treatment. Whole blood from each participant was sampled in acid-citrate dextrose blood collection tubes for plasma separation and PAXgene blood RNA tubes for RNA extraction. Blood tubes were immediately stored at –20°C and were transported from SNMH, Leh, to CSIR IGIB, Delhi, in cold chain for further molecular analysis.

### Clinical evaluation for HAPE diagnosis

As schematically represented in Figure 1B, the diagnosis of HAPE was confirmed by physicians from SNMH, Leh, who have specialization in HA illnesses. Clinical presentation for HAPE included symptoms such as chest tightness or pain, cough, and dyspnea at rest, along with physical signs like pulmonary rales, cyanosis, tachycardia, tachypnea, and reduced peripheral oxygen saturation (SpO₂), indicating hypoxemia. Based on the clinical signs and chest X-ray images, HAPE patients were further segregated into mild, moderate, and severe. Mild symptoms include dyspnea on exertion, dry cough, and fatigue while moving uphill. Clinical signs include heart rate (HR) <100 beats per minute, respiratory rate (RR) <20 breaths per minute, dusky nail beds or exertional desaturation, and localized crackles, if any. Chest X-ray showing minor exudate involving less than 25% of one lung. Moderate symptoms include dyspnea at rest, weakness, fatigue on level walking, and a raspy cough. Clinical signs include HR between 100 and 110 beats per minute, RR between 20 and 28 breaths per minute, and crackles present. Chest X-ray showed some infiltrate involving 50% of one lung or a smaller area of both lungs. Severe symptoms include dyspnea at rest, extreme weakness, orthopnea, and productive cough. Clinical signs include HR of more than 110 beats per minute, RR of more than 28 breaths per minute, nail bed cyanosis, bilateral crackles, and white or pink frothy sputum. Chest X-ray showing bilateral infiltrates more than 50% of each lung.

### RNA extraction and quality assessment from blood samples

RNA was extracted using PAXgene blood RNA kit (catalogue number:762174, PreAnalytiX, Switzerland) according to manufacturer instructions. RNA yield was measured by Qubit RNA BR Assay Kit (Thermo Fisher Scientific, U.S.A.). The purity of RNA was measured by nanodrop (Thermo Fisher, U.S.A.), and its integrity was analyzed by measuring the RNA integrity number (RIN) (Thermo Fisher, U.S.A.). RNA samples after extraction were stored at –80°C until library preparation.

### RNA library preparation and sequencing

RNA samples with RIN greater than 7 and a concentration of 500 ng were used for library preparation. The steps involved in RNA sequencing are illustrated in Figure 1C. Briefly, the process included ribosomal RNA (rRNA) removal, RNA fragmentation, complementary DNA (cDNA) synthesis, adapter ligation, library enrichment, and quantification. Ribosomal RNA, including 5S, 16S, and 23S rRNA, was removed from the samples using the QIAseq FastSelect Epidemiology Kit (Qiagen, Germany). First- and second-strand cDNA synthesis was performed via reverse transcription of the RNA insert. Following library preparation, PCR enrichment was performed to amplify the target library using the QIAseq Stranded RNA Library Kit (Qiagen, Germany). The library concentrations were normalized with the QIAseq Normalizer Kit (Qiagen, Germany). Library quality was assessed by determining size and molarity using a high-sensitivity DNA Agilent chip on the Agilent 2100 Bioanalyzer (Agilent Technologies). Equimolar libraries were pooled for sequencing with an Illumina pooling calculator. The pooled library was sequenced on the NovaSeq 6000 platform using a 2 × 150 bp paired-end read length, generating 20 million reads per library for data analysis.

### Quality validation and data processing

The sequenced data generated in CBCL format from the NovaSeq 6000 platform were demultiplexed to generate FASTQ files using the bcl2fastq tool. The raw FASTQ files are available on the Gene Expression Omnibus (accession number GSE291471) [27].The preliminary quality checking of raw sequencing reads was performed using the FastQC tool. The low-quality bases and adapters were removed using the fastp tool. The reads were further mapped to the human reference genome (GRCh38.p14) using a splice-aware tool, STAR aligner (v 2.7.11b). PCR and optical duplicate sequences were discarded from the BAM file using GATK’s MarkDuplicates. A postalignment quality check of the data was done using the Qualimap tool, and samples with less than 60% mapping rate were removed from the analysis.

### Identification of differentially expressed genes (DEGs)

As outlined in Figure 1D, the read summarization step was carried out with the FeatureCounts tool based on the GENCODE v45 GTF file, and the read counts for each gene were further taken for DE analysis. In DEG analysis, the read count matrix was imported into R, and genes with read counts less than 10 for a minimum number of samples in a group were removed from the matrix before proceeding with the modeling procedure. In the design matrix of DESeq2, categorical variables such as gender, body mass index (BMI), age, and batch effect were used as covariates along with the disease status as the outcome, and the Wald test was used to compare the differences in expression levels of genes between two groups. The genes were considered significantly DE when the associated Benjamini & Hochberg adjusted *P*-value was <0.05 and Log₂ fold change (Log₂FC) > 1.5 and < -1.5 for up-regulated and down-regulated genes, respectively.

### Functional and pathway enrichment analysis

The functional enrichment analysis was carried out with the gene set enrichment analysis method (GSEA) using the clusterProfiler R package. The gene list based on their Log₂FC was subjected to GSEA, where a list of pre-defined gene sets was tested for significance. The datasets used in the study are the molecular signatures database (msigDB) and Kyoto Encyclopedia of Genes and Genomes (KEGG) pathways. Gene ontology (GO) enrichment was employed to determine the enrichment of GO terms in biological processes (BPs), molecular functions (MFs), and cellular components (CCs).

### Construction of a protein–protein interaction (PPI) network and selection of hub genes

STRING database constructed PPI network using the common DEGs (Figure 1D). Cytoscape software was used to visualize the network. Moreover, the hub genes were selected using CytoHubba, another plug-in of CytoScape, according to the number of associations with other genes in the PPI network [28–30]. Eleven common algorithms (MCC, MNC, DMNC, Degree, Closeness, Radiality, Stress, EPC, Eccentricity, bottleneck, and betweenness) were used for evaluating and selecting hub genes. The top 10 genes in each algorithm were further overlapped, and the hub genes were selected.

### Immune cell abundance landscape

Immune cell abundance in the study groups was explored by a reference-based cell type deconvolution approach, the CIBERSORTx algorithm. Read count data were normalized with the DESeq2 package and used as the mixture file for the cell type deconvolution analysis. The reference signature matrix file was the LM22 microarray datasets provided by the CIBERSORTx group, which had weights for 547 genes of 22 cell types [31]. A batch correction was applied before the analysis to remove the batch-wise differences between the mixture and signature matrix files. The permutations were set to 1000 times along with absolute score parameters. According to the scores, a relative abundance of the cell types was calculated for each of the 22 cell types.

### Olink-based targeted protein profiling

Briefly, as outlined in Figure 1E a targeted 48-cytokine panel comprising interleukins, chemokines, cytokines, and other factors quantified the levels of 45 cytokines (Supplementary Figure S1A). The assay was performed on plasma samples using a proximity extension assay, following the manufacturer’s instructions (Olink, Thermo Fisher Scientific, U.S.A.) on Olink Signature Q100 System. Plasma was obtained by centrifuging blood samples at 1,000–2,000×g for 10 min. In brief, paired cDNA-tagged antibodies specific to the target serum proteins enabled the hybridization of their corresponding DNA oligonucleotides, which were then extended by DNA polymerase. Protein levels were quantified using real-time PCR. The normalized protein expression (NPX) values were derived from the Ct values of the real-time PCR run (RT PCR) and subsequently transformed to the log2 scale.

### Identification of differentially expressed lncRNA

Figure 1F shows a basic framework for the lncRNA profiling that filtered out lncRNA genes based on gene biotypes from GENCODE v45 GTF after differential gene expression. The genes were considered significantly DE when the associated Benjamini & Hochberg adjusted *P*-value was <0.05 and Log₂FC > 1.5 and < -1.5 for up-regulated and down-regulated genes, respectively.

### Weighted gene co-expression network analysis of mRNA and lncRNA pairs

The co-expression network analysis of mRNA and lncRNA pairs was carried out with an R package weighted gene co-expression network analysis (WGCNA) in a variance stabilization transformed (vst) count data (Figure 1F). To construct the co-expression network, Pearson’s correlation for all genes was calculated and transformed into an adjacency matrix by raising the correlation coefficient to a soft thresholding power of beta = 14, which was chosen based on the scale-free topology criterion. Then, a topological overlap matrix (TOM) was calculated from the adjacency matrix and converted into a dissimilarity TOM to produce a tree using the hierarchical clustering method. A dynamic tree-cutting function was used to pick the modules. For each module, the genes are summarized into eigengene, the first principal component of the expression data of genes present in that module. Further, the clusters with similar expression profiles were merged, leading to the final modules. Module trait analysis was carried out, in which we encoded our controls as the reference and HAPE patients as the disease group, and based on that, the correlation matrix was calculated. The genes of the modules having positive correlations with HAPE have been processed for the analysis. The interaction network was constructed using two key metrics: module membership (MM) and gene significance (GS). MM measures a gene’s association with a module, with a threshold correlation coefficient > 0.8, indicating potential hub genes, whereas GS reflects a gene’s biological relevance to the trait, also requiring a correlation coefficient >0.8. The visualization of the co-expression network is done using the Cytoscape software, and the selection of hub genes is made using the Cytohubba plugin.

### Real-time PCR

Total RNA isolated from the blood samples was reverse transcribed using a RevertAid first-strand complementary DNA (cDNA) synthesis kit (Thermo Fisher, U.S.A.). The reaction was performed at 42°C for 60 min, followed by the termination step at 70°C for 5 min. cDNA product was diluted at the ratio of 1:5 with nuclease-free water and was stored at −20°C until gene expression analysis. A 10 µl reaction volume per well having SYBR Green master mix, gene-specific primer pairs, and cDNA template was prepared for gene expression analysis through real-time PCR (Bio-Rad CFX96, USA). PCR conditions followed for the assay were 95°C denaturation for 3 min, followed by 40 cycles of 95°C for 3 sec and 60°C for 20 sec. The primer details of the genes are presented in Supplementary Table S1. The 2^-ΔΔCt method was used for fold change calculation of gene expression between the two groups, where ΔCt = Ct GOI – Ct HKG, (GOI is the gene of interest and HKG is the selected housekeeping gene) and mean ΔΔCt = (Δ Ct (Experimental group) −Δ Ct (Control)).

### Statistical analysis

The R programming language was used for statistical analysis and plotting. An individual gene comparison among groups was done with Welch’s *t*-test. The Wald test compared the differences in expression levels of genes between the two groups. Correlation analysis in the study was done using Spearman correlation, and Pearson’s correlation was used for WGCNA. The clinical parameters and cytokine profiles were analyzed using GraphPad Prism software (version 9.0.2, GraphPad Software U.S.A.) and are reported as mean ± SD. The ROC curve analysis was performed to illustrate the sensitivity and specificity of the hub genes.

## Results

### Clinical and anthropometric assessment of the study participants

A total of 95 participants were recruited for the study. However, a few samples were removed due to their failure at quality validation and data processing steps. After removing the samples, 19 controls, 15 mild, 13 moderate, and 14 severe HAPE patients, and 22 HLs were considered for clinical profile, RNA sequencing analysis for differential gene expression profile, targeted protein panel, and differential lncRNA profile in the study (Figure 1A–F). The demographic-based analysis highlights significant differences in age, gender, and BMI between the HAPE group and the controls group (Table 1). Therefore, these variables were considered covariates in all subsequent analyses. Clinical parameters assessments showed a significant decrease in SpO_2_ percentage in the HAPE group compared with controls (58.5 ± 14.9 vs 92.2 ± 2.5; *P<*0.0001*;* Table 1; Supplementary Figure S2Ai). The HAPE group also exhibited higher RR, PR, and mean arterial pressure (MAP) in mmHg relative to controls (23.7 ± 3.4 vs 19.3 ± 1.2, *P<*0.0001; 112 ± 18.1 vs 90.3 ± 14.9, *P<*0.0001; and 96.5 ± 13.6 vs 91.2 ± 6.5, *P=*0.0019*,* respectively; Table 1; Supplementary Figure S2 Aii-iv). Similarly, systolic blood pressure (SBP) increased in the HAPE group compared with controls (129 ± 17.2 vs 118.5 ± 7.4, *P=*0.0076*,* Table 1). HAPE severity showed a consistent decrease in SpO_2_ percentage and a consistent increase in RR, PR, SBP, and MAP (Table 1) among mild, moderate, and severe cases of HAPE, respectively. No significant difference in all these parameters was found between HLs and controls. The assessment of the CBC profile, which included 14 parameters such as neutrophils (Neu), white blood cells (WBC), monocytes (Mon), red blood cells (RBC), immature granulocytes (IMG), platelets (PLT), platelet-large cell ratio (P-LCC), plateletcrit (PCT), hematocrit (HCT), and Hemoglobin (HGB) showed that the levels of Neu and IMG were significantly high in the HAPE group when compared with controls (Supplementary Figure S2 Bi and ii). Conversely, the levels of RBC, HCT, PLT, HGB, PCT, and P-LCC were significantly lower in the HAPE group (Supplementary Figure S2 Biii-viii). Furthermore, in HLs, the levels of PCT, P-LCC, WBC, and Mon were significantly lower compared with controls (Supplementary Figure S2 Bvii- x).

**Table 1:** Demographic and clinical characteristics of the healthy HA sojourners (controls), high-altitude pulmonary edema (HAPE) patients, and long-term HA residents (HLs)

Demographic or Clinical characteristics		Controls (25)	HAPE (45)	HAPE classification			HLs (25)	*P* values	
				Mild (15)	Moderate (15)	Severe (15)		Control vs HAPE	Control vs HLs
Age, years		30.8 ± 7.4	38.4 ± 12.2	39.6 ± 12.8	41.4 ± 12.6	34 ± 9.5	29.7 ± 5.1	0.0069	0.534
Gender	Male	25	34	11	9	24	20	-	-
	Female	0	11	4	6	1	5	-	-
BMI		23.9 ± 3.5	28.7 ± 5.3	27.1 ± 5.6	29.2 ± 4.2	29.8 ± 5.6	22.8 ± 3.1	< 0.0001	0.174
SpO_2_ levels, _%_		92.2 ± 2.5	58.5 ± 14.9	67.2 ± 11.5	59.4 ± 11.9	49 ± 15^***^	92.4 ± 2.8	< 0.0001	0.958
RR, breath/min		19.3 ± 1.2	23.7 ± 3.4	22.4 ± 2.7	22.9 ± 2.6	25.9 ± 3.6^***^	18.7 ± 1.3	< 0.0001	0.070
PR, beats/min		90.3 ± 14.9	112 ± 18.1	100 ± 13.7	111 ± 14.1	123.7 ± 18.1^**^	84.7 ± 10.1	< 0.0001	0.110
MAP, mmHg		91.2 ± 6.5	96.5 ± 13.6	91.6 ± 6.9	94.8 ± 15.5	106 ± 13.5^*^	94.4 ± 2.5	0.0019	0.222
SBP, mmHg		118.5 ± 7.4	129 ± 17.2	119 ± 12.6	125 ± 14.5	143 ± 14.8^***^	119.5 ± 7.3	0.0076	0.568
DBP, mmHg		77.5 ± 9.5	80 ± 16.2	77.9 ± 7.77	78.2 ± 18.4	84 ± 16.7	80.5 ± 6.8	0.4695	0.257

Data are presented as mean ± SD and are compared among the groups by unpaired Student’s *t*-test.

*P is the *P* value among the mild, moderate, and severe categories calculated using an ANOVA test. Here, *P<0.05, **P<0.001, ***P<0.0001.

DBP = diastolic blood pressure. MAP = mean arterial pressure. PR = pulse rate. RR = Respiratory rate. SBP = systolic blood pressure. SpO2 = Peripheral oxygen saturation.

### Differential gene expression profile and functional enrichment analysis of HAPE patients demonstrate enrichment of inflammatory pathways

**Figure 2:**
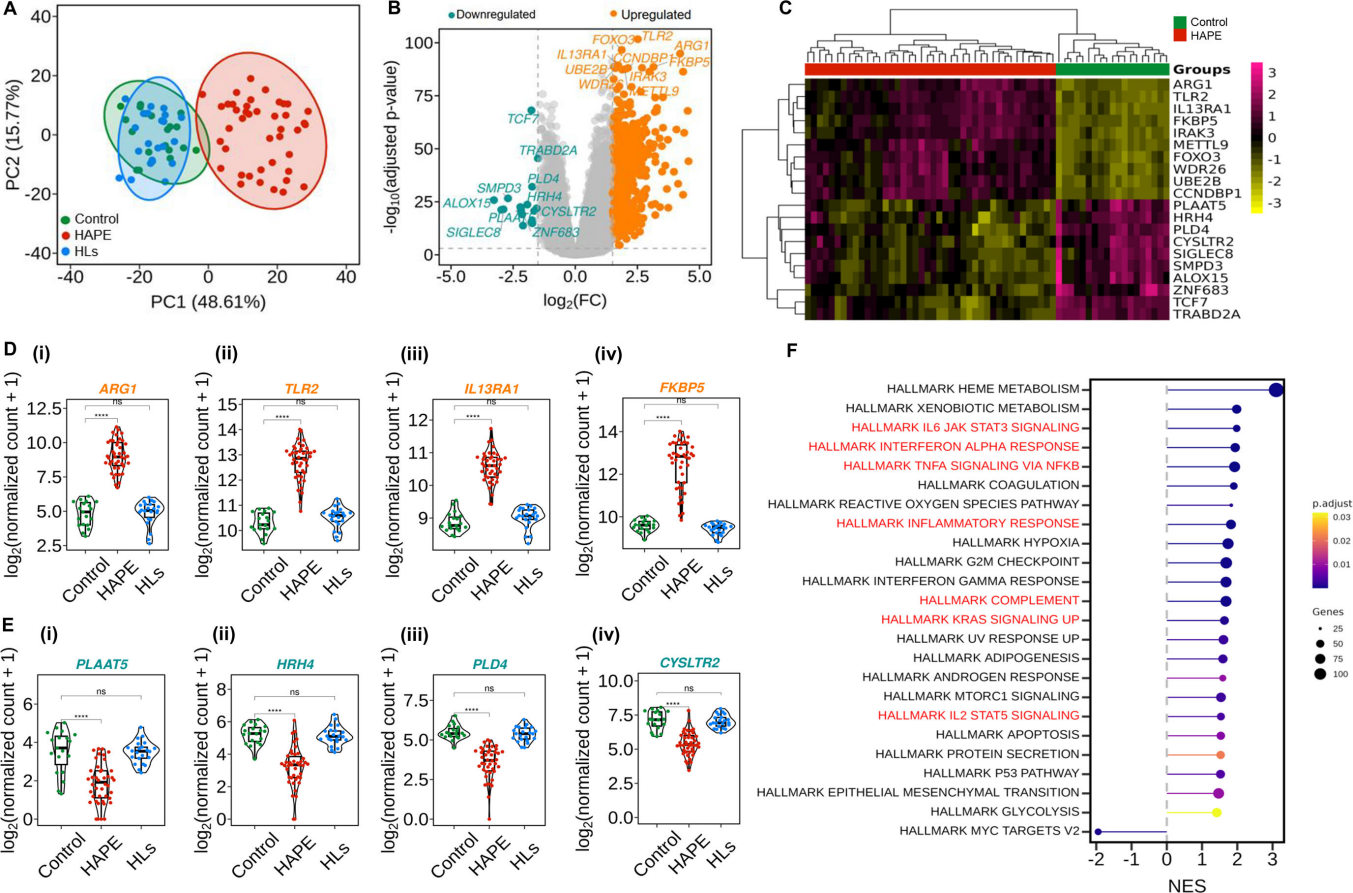
Differential gene expression profile and functional enrichment analysis of HAPE patients demonstrate enrichment of inflammatory pathways. (**A**) PCA represents three clusters for the three study groups based on the expression profile. The PC1 axis explains 48.61% variance, and PC2 explains 15.77% variance. (**B**) Each point in the volcano plot represents a gene, with the *x*-axis showing the log_2_FC to indicate the magnitude of gene expression changes and the *y*-axis showing the negative log_10_ transformation of the adjusted *P* value. The orange dots indicate up-regulated genes, the green dots indicate down-regulated genes, and the gray dots indicate nonsignificant genes. (**C**) Heatmap illustrates patterns of gene expression of the top 10 up-regulated and down-regulated genes, with the color scale representing the intensity of gene expression. The Beeswarm plot represents the median normalized count of (**D**) the top 4 up-regulated genes and (**E**) the top 4 down-regulated genes in controls (*n* = 19), HAPE patients (*n* = 42), and HLs (*n* = 22). (**F**) pathway enrichment analysis of DEGs by msigDB hallmark pathways. Each stick represents the enrichment level of a specific pathway, with the length of the bar indicating the enrichment score. The depth of the color represents the log of the *P* value, and the size of the ball represents the number of genes involved in the pathway. The dots in the PCA and beeswarm plots represent the number of samples, and here *P<0.05, **P<0.01, ***P<0.001, ****P<0.0001*,* and ns is nonsignificant.

Principal component analysis (PCA) of the top 500 highly varying genes revealed distinct clustering among the groups. The HAPE group clustered separately from control and HLs along the PC1 and PC2 axes, which accounted for 48.61% and 15.77% variance, respectively (Figure 2A). Among the 515 significant DEGs, 497 genes were up-regulated, and 18 genes were down-regulated (Figure 2B and Supplementary Table S2). The heatmap illustrates the hierarchical clustering of the top 10 up-regulated and down-regulated genes based on *P*-adjusted value (Figure 2C and Table 2). Beeswarm plot shows the expression levels in log_2_-normalized counts of the top 4 up-regulated and down-regulated genes in the study groups (*P*<0.05; Figure 2Di-iv, and Ei-iv). Next, the top 5 up-regulated and down-regulated genes were validated using real-time PCR (Supplementary Figure S3 Ai-v, and Bi-v). Functional enrichment analysis was performed to understand the biological functions of the genes. The msigDB analysis revealed significant enrichment of hallmark inflammatory pathways including IL6/JAK/STAT3 signaling, interferon alpha response, TNFa signaling via NF-κB, inflammatory response, complement response, kras signaling, IL2/STAT5 signaling, and hallmark hypoxia (*P*<0.05; Figure 2F). KEGG analysis highlighted neutrophils extracellular trap formation, complement and coagulation cascades, and Nod-like receptor signaling pathways (Supplementary Figure S4A). Similarly, GO analysis highlighted the enrichment of inflammation-related pathways in the BPs, CCs, and MFs-based analysis (Supplementary Figure S4 B-D). A similar DEGs analysis was performed in the mild, moderate, and severe HAPE cases compared with controls (Supplementary Figure S5, Supplementary Figure S6 A-F; Supplementary Table S3-5). Functional enrichment analysis using msigDB and KEGG was also analyzed in the segregated HAPE cases compared with controls (Supplementary Figure S6 G-L).

**Table 2:** The top 10 differentially expressed mRNAs between HAPE patients vs controls

Up-regulated genes	log_2_FC	Adjusted *P* value	Down-regulated genes	log_2_FC	Adjusted *P* value
ARG1	4.219364599	6.24E−42	PLAAT5	−2.209571661	1.65E−10
TLR2	2.517274855	6.81E−45	HRH4	−1.929906736	5.31E−11
IL13RA1	1.725984471	1.54E−39	PLD4	−1.735417411	1.14E−14
FKBP5	3.147934191	3.16E−39	CYSLTR2	−1.552683541	2.95E−10
IRAK3	2.684922508	4.94E−39	SIGLEC8	−2.889104757	4.86E−10
METTL9	1.995442124	9.60E−39	SMPD3	−2.70615125	2.75E−12
FOXO3	1.868356509	1.06E−42	ALOX15	−3.27334016	6.28E−12
WDR26	1.616266181	5.83E−39	ZNF683	−2.183173804	4.96E−10
UBE2B	1.553263708	2.87E−39	TCF7	−1.764162649	2.55E−30
CCNDBP1	2.120981764	5.34E−39	TRABD2A	−1.503197322	1.77E−20

The log_2_FC represents the magnitude and direction of expression, with positive values indicating up-regulation and negative values indicating down-regulation. The adjusted *P* values were computed using the Benjamini–Hochberg method to control the false discovery rate across multiple comparisons. Genes with *P* adjusted < 0.05 were considered significantly differentially expressed.

### PPI network highlights genes associated with inflammation, and the immune cell abundance landscape recognizes altered immune cell abundance

**Figure 3:**
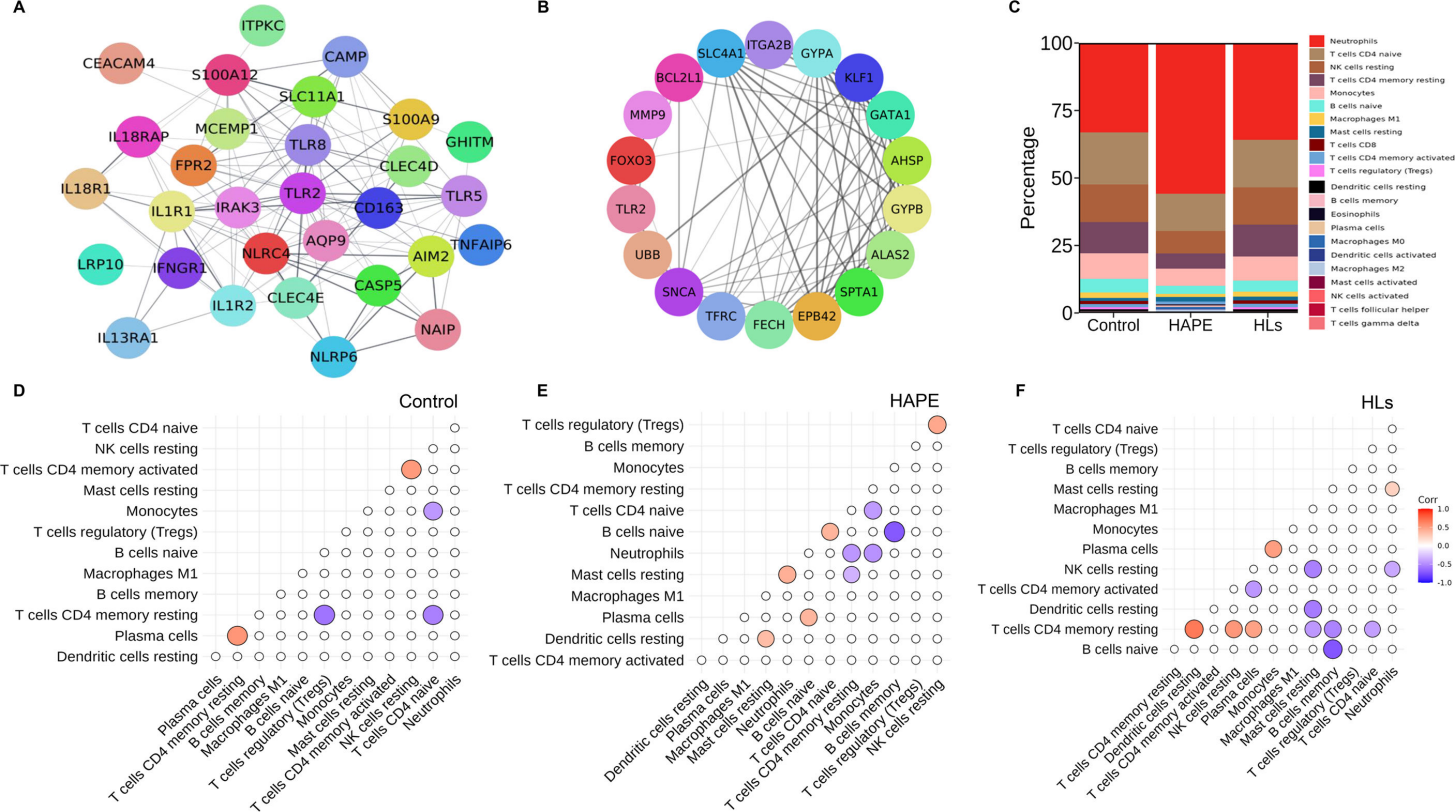
The protein-protein interaction network highlights genes involved in inflammation, and the immune cell abundance landscape recognizes altered immune cell abundance. (**A**) Key inflammatory cluster obtained from PPI network (**B**) The PPI network of 18 hub genes was identified after analyzing the eleven algorithms of cytohubba in Cytoscape. (**C**) The plot illustrates the percentage abundance of 22 different immune cell types across the three study groups. Spearman’s rank correlation depicting correlations between infiltrated immune cells in (**D**) Controls (*n* = 19), (**E**) HAPE patients (*n* = 42), and (**F**) HLs (*n* = 22). Here, the circle represents the correlation value in the plot.

To elucidate the interactions among differentially expressed genes, a PPI network was constructed for the top 515 significant DEGs in the HAPE group compared with controls using Cytoscape and the plug-in string (Supplementary Figure S7). The key inflammatory cluster obtained from the PPI network is presented in Figure 3A. The top 10 hub genes identified using each algorithm in the PPI network (Supplementary Figure S8 and Supplementary Table S6) were further overlapped, and 18 hub genes were identified (Figure 3B and Table 3). Of the 18 hub genes, the receiver operating curve (ROC) analysis of *TLR2, FOXO3, ALAS2,* and *SPTA1* showed 100% sensitivity and specificity (Supplementary Figure S9 and Supplementary Table S7). The relative percentage abundance of 22 types of immune cells in each sample was quantified and presented using immune cell abundance analysis (Figure 3C). Neutrophils showed the most significant difference, with HAPE patients exhibiting nearly 50% abundance (Figure 3C). The individual scores of significant immune cells in the three groups are presented in Supplementary Figure S 10. Further, pairwise Spearman correlation analysis in cell types with nonzero scores was performed among the cell types within the study groups (Figure 3D–F).

**Table 3:** The hub genes derived from the PPI network between HAPE patients vs controls

Genes	log_2_FC	Adjusted *P* value
GATA binding protein 1 (*GATA1)*	1.616018923	1.18E−12
Ferrochelatase *(FECH)*	2.527447972	2.67E−20
Synuclein alpha *(SNCA)*	1.906456804	2.23E−14
Transferrin receptor *(TFRC)*	1.731907498	3.80E−13
Matrix metalloproteinase 9 *(MMP9)*	2.003084924	2.52E−11
Solute carrier family 4 anion exchanger member 1 *(SLC4A1)*	2.406722669	1.39E−17
BCL2 like 1 *(BCL2L1)*	2.074891415	1.17E−18
Krüppel like factor 1 *(KLF1)*	1.740932832	4.48E−08
Erythrocyte membrane protein band 4.2 *(EPB42)*	2.598122913	2.12E−18
Forkhead box O3 *(FOXO3)*	1.868356509	1.06E−42
Glycophorin A *(GYPA)*	3.49533689	9.40E−18
Glycophorin B *(GYPB)*	2.841071065	7.71E−17
Toll-like receptor 2 *(TLR2)*	2.517274855	6.81E−45
Aminolevulinate synthase 2 *(ALAS2)*	3.103795425	1.47E−22
Integrin alpha IIb *(ITGA2B)*	1.675051736	1.93E−07
Ubiquitin B *(UBB)*	2.057976683	4.70E−12
Alpha hemoglobin stabilizing protein *(AHSP)*	3.72676422	2.75E−24
Spectrin alpha, erythrocytic 1 *(SPTA1)*	2.738075355	1.49E−22

The table represents the log2 fold changes and adjusted *P* values of hub genes derived from the PPI network. The log_2_FC represents the magnitude and direction of expression, with positive values indicating up-regulation and negative values indicating down-regulation. The adjusted *P* values were computed using the Benjamini–Hochberg method to control the false discovery rate across multiple comparisons. Genes with *P*-adjusted < 0.05 were considered significantly differentially expressed.

### Targeted plasma cytokine profiles corroborate with the whole transcriptomic outcomes

**Figure 4:**
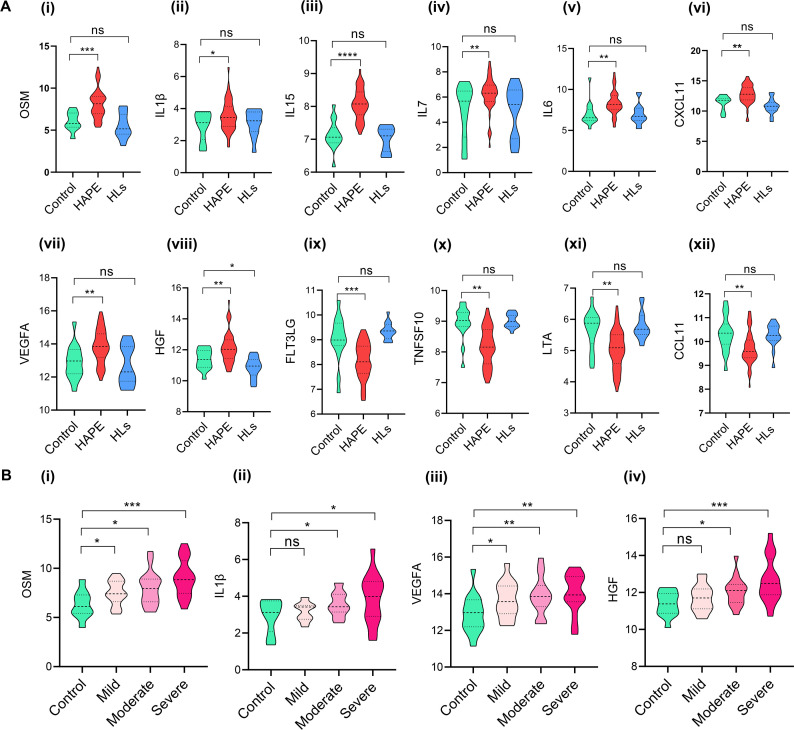
Targeted plasma cytokine profiles represent higher levels of inflammatory cytokines. (**A**) The violin plots represent median value of the cytokine levels expressed as normalized protein expression in controls (*N* = 15), HAPE patients (*n* = 45) and HLs (*n* = 15) for (**i**) OSM, (ii) IL1β, (iii) IL15, (iv) IL7, (**v**) IL6, (vi) CXCL11, (vii) VEGFA, (viii) HGF, (ix) FLT3LG, (**x**) TNFSF10, (xi) LTA, (xii) CCL11. (**B**) The violin plots represent the median value of the cytokine levels in the mild (*n* = 15), moderate (*n* = 15), severe (*n* = 15) HAPE groups as compared with controls (*n* = 15) for (**i**) OSM, (ii) IL1β, (iii) VEGFA, and (iv) HGF. Here, *P<0.05, **P<0.01, ***P<0.001, ****P<0.0001 and ns is nonsignificant.

The targeted 48-cytokine panel comprising of interleukins, chemokines, cytokines, and other factors quantified the levels of 45 cytokines (Supplementary Figure S1 A). Out of the 45 cytokines analyzed, 37 had more than a 75% yield (Supplementary Figure S1 B). The levels expressed in NPX for Oncostatin-M (OSM), Interleukin-1 beta (IL1β), Interleukin-15 (IL15), Interleukin-7 (IL7), Interleukin-6 (IL6), C-X-C motif chemokine 11 (CXCL11), Vascular Endothelial Growth Factor A (VEGFA), and Hepatocyte Growth Factor (HGF) were significantly higher in the HAPE group compared with controls (Figure 4Ai–viii). Conversely, the levels expressed in NPX for Fms-related tyrosine kinase 3 ligand (FLT3LG), Tumor Necrosis Factor Ligand Superfamily Member 10 (TNFSF10), Lymphotoxin-alpha (LTA), and Eotaxin (CCL11) were significantly lower in the HAPE group compared with controls (Figure 4A ix-xii). Among all the molecules, only HGF showed a significant difference in (HLs) compared with controls (Figure 4A viii ). Interestingly, when comparing mild, moderate, and severe HAPE categories to controls, only four cytokines, OSM, IL1β, VEGFA, and HGF, showed a significant increase in levels with disease severity (Figure 4Bi-iv).

**Figure 5:**
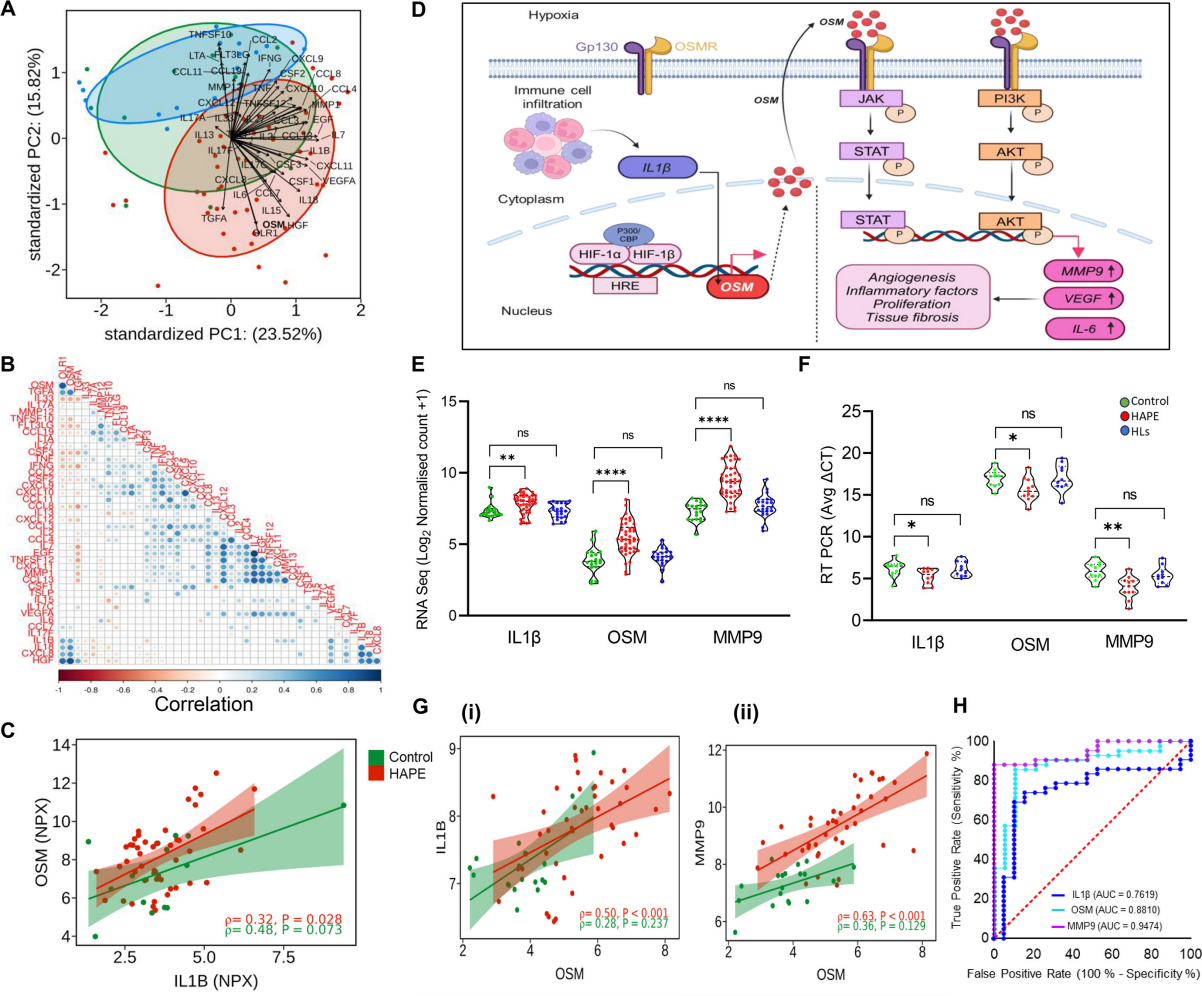
Targeted plasma cytokine profiles corroborated with the whole transcriptomics insights. (**A**) PCA of the 45 cytokines in the three study groups. The dot represents samples, and the arrow represents the magnitude of each cytokine. (**B**) The correlation heatmap represents the correlation pattern between all the cytokines of the panel. (**C**) The scatter plot represents a positive correlation in NPX levels of OSM and IL1β. (**D**) Visual representation of OSM up-regulation and activation of its downstream genes and pathways. **(E**) The violin plot represents the median normalized count calculated by RNA sequencing of 1L1β, OSM, and MMP9 in the three study groups in each sample. (**F**) The violin plot is representative of the average delta (Δ) ct calculated by RT PCR of 1L1β, OSM, and MMP9 in controls (*n* = 10), HAPE patients (*n* = 10) and HLs (*n* = 10). (**G**)The scatter plot represents a positive correlation in (**i**)OSM and IL1β cytokine, (ii) OSM and MMP9. (**H**) ROC of the IL1β, OSM, and MMP9 genes with AUC of each gene. Here, *P<0.05, **P<0.01, ***P<0.001, ****P<0.0001 and ns is nonsignificant.

The PCA of the cytokines in the three study groups showed an underlying similarity between the controls and HLs. In contrast, the HAPE group exhibited a 23.52% variance in PC1 and 15.82% variance in PC2 axes, primarily influenced by OSM, Oxidized Low-Density Lipoprotein Receptor 1 (OLR1), HGF, and Pro Transforming Growth Factor Alpha (TGFA) (Figure 5A). OSM and OLR1 also had the highest pairwise correlation pattern in the diagonal correlation matrix (Figure 5B). Additionally, OSM and IL1β showed a significant positive correlation of 0.32 in Spearman correlation analysis (Figure 5C), supporting the mechanism where IL1β release induces OSM expression (Figure 5D). Notably, the downstream genes activated by OSM-induced signaling include *MMP9, VEGFA*, and *IL-6,* which play essential roles in extracellular matrix degradation, angiogenesis, and the inflammatory response (Figure 5D). The expression level of *IL1β, OSM,* and *MMP9,* validated by real-time PCR, was significantly high in the HAPE group as compared with controls (Figure 5E and F). Spearman correlation analysis of gene expression data for *IL1β, OSM,* and *MMP9* in HAPE patients showed significant positive correlations of 0.50 between *IL1β* and *OSM* and 0.63 between *MMP9* and *OSM* (Figure 5Gi and ii). ROC analysis demonstrated an AUC of 0.7619 for *IL1β*, with 76.19% sensitivity and 73.68% specificity. *OSM* exhibited an AUC of 0.881, with 90.48% sensitivity and 89.47% specificity, while *MMP9* had an AUC of 0.9474, with 97.62% sensitivity and 94.74% specificity (*P*<0.05, Figure 5H).

### Identification of differentially expressed lncRNA and weighted gene co-expression network analysis for mRNA and lncRNA pairs

**Figure 6:**
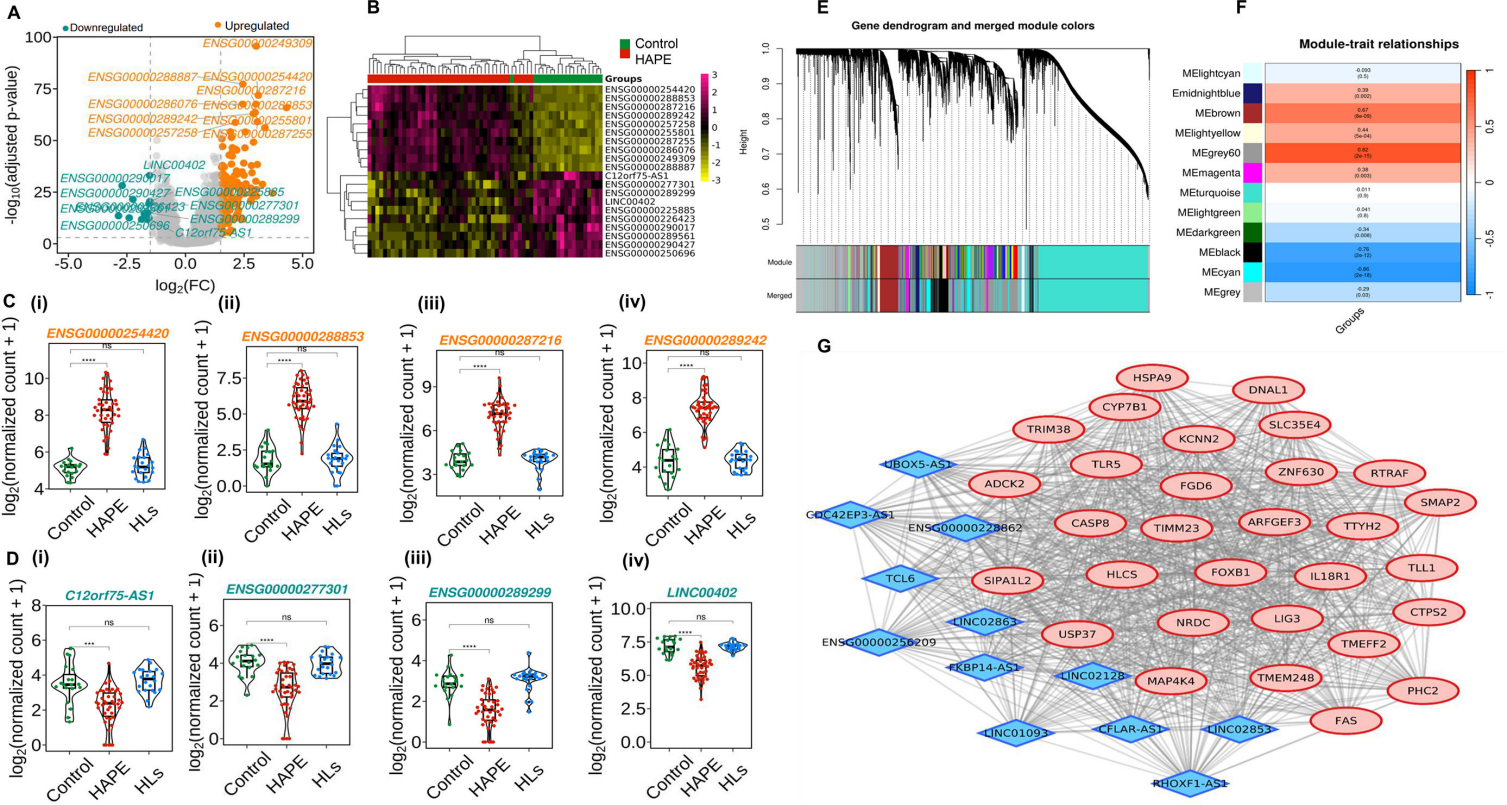
Identification of differentially expressed long noncoding RNA and weighted gene co-expression network analysis of mRNA and lncRNA pairs. (**A**) Each point in the volcano plot represents a gene, with the *x*-axis showing the log_2_FC to indicate the magnitude of gene expression changes and the *y*-axis showing the negative log_10_ transformation of the adjusted *P* value. The orange dots indicate up-regulated genes, the green dots indicate down-regulated genes and the grey dots indicate nonsignificant genes. (**B**) Heatmap illustrates patterns of gene expression of the top 10 up-regulated and down-regulated LncRNA, with the colour scale representing the intensity of gene expression. The beeswarm plot represents the median normalized count of (**C**) the top 4 up-regulated genes, and (**D**) the top 4 down-regulated genes in controls (*n* = 19), HAPE patients (*n* = 42) and HLs (*n* = 22). (**E**) The co-expressed genes in WGCNA were divided into various gene modules using hierarchical clustering dendrograms, and here, different colours indicate the corresponding modules. (**F**) Each gene module’s associations with HAPE patients are shown visually through the heatmap, and here, positive and negative signs are indicators of positive and negative association with the module. (**G**) The hub genes associated with the grey60 module are presented in the network using a cystoscope; the light red colour presents the mRNA, and the blue colour presents the lncRNA. Here, *P<0.05, **P<0.01, ***P<0.001, ****P<0.0001, and ns is nonsignificant.

Out of 172 differentially expressed lncRNAs, 159 were up-regulated, and 13 were down-regulated in the HAPE group compared with controls (Figure 6A and Supplementary Table S8). Heatmap illustrates the hierarchical clustering of the top 10 up-regulated and down-regulated lncRNAs based on *P*-adjusted values (Figure 6B and Table 4). Beeswarm plots depict the expression levels in log_2_ normalized counts of the top 4 up-regulated and down-regulated lncRNAs in the study groups (Figure 6Ci-iv and Di-iv). The expression levels of the top 5 up-regulated and down-regulated lncRNAs were validated using real-time PCR (Supplementary Figure S11 A and B).

**Table 4:** The top 10 differentially expressed lncRNAs between HAPE patients vs controls

Up-regulated genes	log_2_FC	Adjusted *P* value	Down-regulated genes	log_2_FC	Adjusted *P* value
ENSG00000254420	3.107628161	6.17E−32	C12orf75-AS1	−1.740277686	1.60E−06
ENSG00000288853	4.311840906	2.54E−29	ENSG00000277301	−1.561609463	3.89E−09
ENSG00000287216	3.012811453	4.51E−30	ENSG00000289299	−1.614368616	3.21E−07
ENSG00000289242	2.894304995	4.62E−28	LINC00402	−1.531439203	4.25E−15
ENSG00000257258	2.125667024	3.12E−26	ENSG00000225885	−1.523607151	1.28E−09
ENSG00000255801	2.974475987	2.50E−28	ENSG00000226423	−1.754446628	4.19E−07
ENSG00000287255	3.069254564	2.20E−26	ENSG00000290017	−2.705659561	5.66E−13
ENSG00000286076	2.45147806	4.51E−30	ENSG00000289561	−2.861107132	1.32E−06
ENSG00000249309	3.016852597	2.87E−42	ENSG00000290427	−2.247019886	4.50E−10
ENSG00000288887	2.449496763	2.70E−34	ENSG00000250696	−2.405193099	3.53E−06

The log_2_FC represents the magnitude and direction of expression, with positive values indicating up-regulation and negative values indicating down-regulation. The adjusted *P* values were computed using the Benjamini–Hochberg method to control the false discovery rate across multiple comparisons. Genes with *P*-adjusted < 0.05 were considered significantly differentially expressed.

A weighted gene co-expression network was constructed using WGCNA for the HAPE group and controls. Hierarchical clustering grouped genes into various modules based on co-expression patterns (Figure 6E). Module-trait analysis identified five modules significantly correlated with the HAPE status (Figure 6F). The module with the highest correlation coefficient (*R* = 0.82, *P*=2e-15) was subjected to overrepresentation analysis and was enriched for terms related to immune response, leukocyte cell-cell adhesion, and ncRNA processing (Supplementary Figure S11 C). Cytohubba analysis identified 42 hub genes based on degree, betweenness, and closeness (Figure 6G). Of these hub genes, 12 were lncRNAs and 30 were protein-coding genes (Table 5). The known predicted functions and disease association of these lncRNAs are presented in Supplementary Table S9 [32–43]. Among these, six genes, long intergenic nonprotein coding RNA 1093 (*LINC01093*), interleukin 18 receptor 1 (*IL18R1*), signal-induced proliferation-associated 1 like 2 (*SIPA1L2*), Toll-like receptor (*TLR5*), small ArfGAP2 (*SMAP2*), and polyhomeotic homolog 2 (*PHC2*), were found to be differentially expressed in the HAPE group compared with controls.

**Table 5:** The hub genes derived from WGCNA between HAPE patients vs controls

Genes	log_2_FC	Adjusted *P* value	lncRNA/mRNA
LINC01093	3.285945213	3.12E−13	lncRNA
LINC02863	0.94868478	2.46E−08	lncRNA
LINC02853	0.858840149	0.346148412	lncRNA
CFLAR-AS1	0.848942574	0.001679449	lncRNA
LINC02128	0.647665561	0.410061011	lncRNA
ENSG00000256209	0.240336607	0.795666369	lncRNA
TCL6	−0.074328129	0.860189519	lncRNA
UBOX5-AS1	−0.101676472	0.863379185	lncRNA
ENSG00000228862	−0.156927977	0.788026847	lncRNA
FKBP14-AS1	−0.363199607	0.099725195	lncRNA
CDC42EP3-AS1	−0.775272401	0.000355049	lncRNA
RHOXF1-AS1	−0.839247219	0.033804968	lncRNA
IL18R1	2.991707163	1.26E−23	mRNA
SIPA1L2	2.207742116	9.95E−30	mRNA
TLR5	2.037521929	9.36E−27	mRNA
SMAP2	1.826455965	2.40E−30	mRNA
PHC2	1.81130081	2.19E−23	mRNA
NRDC	1.094242465	1.95E−34	mRNA
MAP4K4	0.698844767	1.63E−12	mRNA
FAS	0.620236119	7.05E−08	mRNA
KCNN2	0.542515023	0.462094991	mRNA
TLL1	0.519340033	0.486262904	mRNA
ARFGEF3	0.501457041	0.485936121	mRNA
TMEFF2	0.453659552	0.48244945	mRNA
CASP8	0.427734997	4.51E−07	mRNA
TRIM38	0.303299727	0.039181991	mRNA
TMEM248	0.13587663	0.033348171	mRNA
ZNF630	0.0638109	0.66344999	mRNA
TIMM23	0.039795523	0.70408638	mRNA
RTRAF	−0.01288962	0.929006533	mRNA
FGD6	−0.086718569	0.697305896	mRNA
FOXB1	−0.116070316	0.889763251	mRNA
SLC35E4	−0.163621553	0.608646352	mRNA
HSPA9	−0.393684567	5.21E−05	mRNA
USP37	−0.444362394	1.37E−11	mRNA
DNAL1	−0.445900786	0.006533496	mRNA
CTPS2	−0.513348877	7.35E−07	mRNA
ADCK2	−0.618719064	7.84E−06	mRNA
LIG−3	−0.629323977	1.83E−12	mRNA
HLCS	−0.712495646	7.88E−09	mRNA
TTYH2	−0.744163771	5.54E−07	mRNA
CYP7B1	−1.112310388	0.000980363	mRNA

The log_2_FC represents the magnitude and direction of expression, with positive values indicating up-regulation and negative values indicating down-regulation. Adjusted *P* values were computed using the Benjamini–Hochberg method to control the false discovery rate across multiple comparisons. Genes with *P* adjusted < 0.05 were considered statistically significant.

## Discussion

The present study explored the link between hypoxia-induced systemic inflammation and HAPE, a condition affecting sojourners who fail to acclimatize to the HA environment. The study identified key coding and non-coding molecular factors and inflammatory players in HAPE patients compared with acclimatized HAPE-free sojourners and long-term HA natives. The association between inflammation and HAPE development was a central focus, revealing that blood transcriptome profiling provides a comprehensive snapshot of the body’s physiological state. Clinical assessments indicated respiratory distress and impaired oxygenation that worsened with severity, consistent with findings in previous studies [44]. Hematological profiling highlighted immune system activation and hematopoietic impairment in HAPE patients. This was evidenced by an increased neutrophil and immature granulocyte count, suggesting hypoxia-driven inflammation. Previous reports have also noted elevated neutrophil levels in individuals experiencing high-altitude conditions, linking them to acute mountain sickness and HAPE [45,46]. Similarly, studies have shown that hypoxia can inhibit the proliferation and differentiation of RBCs and platelets [47,48] and that inflammatory cytokines can suppress erythropoiesis [49]. Additionally, the elevated WBC count in HAPE patients indicated systemic inflammation and immune activation [50], while the reduced monocyte levels compared with controls suggest their migration from circulation to lung tissues at the site of hypoxic injury [9,51].

Building on our promising clinical data, we advanced to a detailed differential gene expression analysis, which provided comprehensive pathological insights into the development of HAPE. Principal component analysis (PCA) revealed a distinct clustering of HAPE patients compared with the overlapping patterns of controls and high-altitude sojourners, indicating unique gene expression profiles in the patient group. Functional enrichment analysis identified several hallmark inflammatory pathways, including IL-6/JAK/STAT3 signaling, interferon-alpha response, TNF-alpha signaling via NF-κB, inflammatory response, complement activation, Kras signaling, and IL-2/STAT5 signaling. These findings underscore the significant role of inflammatory processes in the pathophysiology of HAPE.

Further analysis of the PPI network revealed key inflammatory cluster along with 18 hub genes that play central roles in both erythropoiesis and inflammation. Genes primarily associated with erythropoiesis, including *GATA1, KLF1, ALAS2, FECH, AHSP, SLC4A1, SPTA1, EPB42, GYPA*, and *GYPB*, are involved in RBC development and function, thereby ensuring effective oxygen delivery under hypoxic stress. Among these genes, *GATA1* shows a direct link to inflammation, as its expression is suppressed by pro-inflammatory cytokines such as *TNF-α* and by inflammasome activation [52,53]. This establishes a direct connection between inflammatory signaling and impaired erythropoiesis. Moreover, *GATA1* also influences macrophage polarization, thereby shaping immune responses [54]. *KLF1, ALAS2*, and *SLC4A1* demonstrate indirect associations with inflammation. *KLF1*, although primarily a regulator of erythroid differentiation, is also connected to hypoxia and stress pathways, which may intersect with inflammatory mechanisms [55]. *ALAS2* and *SLC4A1* contribute to red cell integrity by limiting oxidative stress and, in doing so, can activate cytokine release and inflammatory signaling pathways [56,57]. In contrast, *FECH, AHSP, SPTA1, EPB42, GYPA*, and *GYPB* are predominantly involved in heme biosynthesis, red blood cell maturation, and membrane stability, with no strong evidence directly linking them to inflammatory signaling. Apart from these genes, *TLR2, MMP9, FOXO3*, *BCL2L1*, and *UBB* modulate inflammatory pathways. *TLR2* triggers innate immune activation via NF-κB and MAPK pathways, leading to cytokine release and inflammation [58]; *MMP9*, a matrix metalloproteinase, plays a crucial role in ECM degradation and vascular leakage, contributing to increased pulmonary permeability, a key feature of HAPE [59–61]; *FOXO3*, a transcription factor involved in oxidative stress response and cell survival, suggests an adaptive mechanism against hypoxia-induced damage [62]; *BCL2L1* supports erythroid cell longevity under oxygen deprivation by preventing apoptosis [63]; and *UBB*, through its central role in protein turnover via the ubiquitin proteasome system, may help manage hypoxia-induced cellular stress and inflammation [64]. Interestingly, the ROC curve for *MMP9, TLR2, FOXO3,* and *BCL2L1* highlighted them as potential hub genes as they demonstrate strong diagnostic potential. The increased expression of these genes highlights the relationship between immune response, inflammation, and tissue damage caused by hypoxia in the pathogenesis of HAPE.

Moving ahead, we conducted an immune cell abundance analysis to deconvolute the RNA sequencing data and identify specific immune cell populations and their relative abundance in HAPE patients. This analysis revealed a higher abundance of neutrophils, resting mast cells, and naïve macrophages in HAPE patients. The elevated neutrophil count aligns with our hematological findings, suggesting a consistent immune response. Increased mast cell activity implicates the release of inflammatory mediators that contribute to vascular permeability, a hallmark of HAPE. Meanwhile, the presence of naive macrophages indicates early immune activation, with potential roles in both inflammation and tissue repair [65–67]. Conversely, significantly lower levels of resting NK cells, resting CD4 memory T cells, CD8, and M1 macrophages could suggest an impaired adaptive immune response and an imbalance that favors excessive inflammation over immune resolution. To further extend our transcriptomics data, we analyzed the targeted cytokine profiles in plasma samples across the study groups. The data revealed significant elevations in OSM, IL1β, IL15, IL7, IL6, CXCL11, VEGFA, and HGF in HAPE patients compared with controls. Among these, OSM, IL1β, IL6, and VEGFA are potent mediators of inflammation and endothelial damage, while IL7, IL15, and CXCL11 primarily regulate immune responses [68–70]. Conversely, the levels of FLT3LG, TNFSF10, LTA, and CCL11 were significantly reduced in HAPE patients, suggesting impaired hematopoiesis, dysregulated vascular responses, and defective resolution of inflammation [71]. The progressive increase in OSM, IL1β, VEGFA, and HGF with disease severity further supports their pivotal role in the progression of HAPE. OSM, alongside OLR1, HGF, and TGF, not only emerged as a key contributor explaining variance in PCA that clustered HAPE separately from the two other groups but also exhibited a significant positive correlation with IL1β, indicating their synergistic effects in enhancing the production of pro-inflammatory cytokines [72]. In addition, OSM is predominantly produced by neutrophils [73,74], which were found in higher abundance in HAPE patients in both immune cell abundance and hematological analyses. Interestingly, dexamethasone, which is used as prophylaxis for acute mountain sickness, has been shown to significantly reduce the production of OSM via NFKB signaling cascades [75]. The presence of a hypoxia response element in the upstream region of the OSM gene further underscores the relevance of our findings under hypoxic conditions [76]. Additionally, we observed significant positive correlations between OSM and MMP9, corroborating the OSM-induced activation of the expression of MMP9, VEGFA, and IL6, which are involved in extracellular matrix degradation, angiogenesis, and inflammation via JAK-STAT and PI3K signaling cascade [77–81]. OSM acts as a pro-inflammatory cytokine that binds the gp130/OSMRβ receptor complex on epithelial and stromal cells, activating MAPK/ERK to drive *MMP9* transcription and secretion [79]. It simultaneously weakens epithelial barrier integrity, shown by reduced transepithelial resistance and disruption of occludin-based tight junctions [73]. Through *STAT3* signaling, *OSM* induces chemokines such as CXCL5, enhancing neutrophil recruitment [82]. Neutrophil-derived and resident cell-induced *MMP9* then degrades extracellular matrix proteins (collagen IV, laminin) and cleaves tight junctions, worsening barrier disruption and vascular leakage, as observed in hypoxia-induced models [83]. Collectively, the *OSM–MMP9* axis links inflammatory signaling to barrier breakdown, neutrophil influx, and vascular leak, resembling mechanisms observed in HAPE.

Noncoding RNAs, particularly lncRNAs, play crucial roles in transcriptional, post-transcriptional, and epigenetic gene regulation. In addition to coding differentially expressed genes, our transcriptomics data revealed significantly differentially expressed lncRNAs in HAPE patients. Through WGCNA-based gene co-expression analysis, we identified co-expressed lncRNAs and mRNAs, which formed a gene module exhibiting a strong correlation with immune response, leukocyte adhesion, and ncRNA processing pathways. Interestingly, these co-expressed partners appear to regulate the NF-κB signaling pathway, one of the most enriched pathways in our analysis. Notably, *LINC01093*, a liver-specific lncRNA, has been implicated in diseases such as alcohol hepatitis and hepatocellular carcinoma [32]. *IL18R1*, a key member of the IL-1 receptor family, can activate NF-κB and is linked to acute mountain sickness and asthma [84]. Similar to our study, Li et al. highlighted the significant differential expression of ncRNA, circulatory RNAs (circRNAs), which correlated with clinical indicators in their study, suggesting their potential role in advancing the diagnosis and treatment of HAPE [85].

However, some limitations of the study warrant further consideration. The absence of female participants in the control group represents a limitation in the study. Although field studies often face practical constraints, gender-specific differences in immune regulation and hypoxia adaptation are well-documented and could affect key molecular signatures such as gene expression and cytokine responses [86]. Additionally, bulk RNA sequencing in blood presents an inherent challenge due to sample heterogeneity and variations in blood cell composition. While blood serves as a readily accessible source for biomarker discovery in cardiovascular disease, sepsis, cancer, and metabolic disorders [87–90], the complexity of its cellular composition may mask cell-specific gene expression changes that are critical for understanding disease mechanisms. Furthermore, while blood-based transcriptome sequencing offers valuable insight into systemic immune responses, it may not accurately reflect the immunological dynamics within the pulmonary vasculature or alveoli that drive HAPE, highlighting a gap between systemic sampling and local pathophysiology. However, the subsequent study of this work will focus on investigating the role of peripheral blood mononuclear cells (PBMCs), particularly monocytes and dendritic cells, which are key players at the site of inflammation. Approaches such as single-cell sequencing may provide a more precise understanding of gene expression dynamics in future studies. The magnitude of gene expression changes alone does not sufficiently support the conclusion of limited enrichment in hallmark pathways; therefore, a more targeted analysis of these pathways might be necessary. Furthermore, while inflammation in HAPE patients can be influenced by various factors, including respiratory tract infections (RTI) [91], investigating their specific contributions was beyond the scope of this study. However, imminent studies can explore and validate RTI as a risk factor and may investigate infection-associated inflammation in HAPE. In conclusion, inflammation plays a critical role in the pathogenesis of HAPE, including exacerbating vascular permeability, endothelial dysfunction, and immune activation. Hypoxia-induced stress leads to the excessive activation of innate immune pathways, characterized by increased neutrophil proportion and the up-regulation of inflammatory cytokines such as IL1β, which induces the expression of OSM. These pro-inflammatory mediators further activate signaling pathways like JAK-STAT and PI3K, triggering the up-regulation of downstream genes such as *MMP9* and *VEGFA*. The elevated expression of these genes promotes extracellular matrix degradation, contributing to vascular leakage, edema formation, and inflammation. Additionally, the study reveals impaired adaptive immune responses, suggesting a failure in immune resolution, which contributes to disease progression. Overall, the comprehensive analysis integrating gene expression, immune cell abundance, cytokine profiles, and epigenetic regulation outlined inflammatory responses in HAPE, enabling the selection of targeted treatments and improved management of hypoxia-related illnesses.

## Supplementary material

online supplementary material 1.

online supplementary material 2.

## Data Availability

Data are now available on the Gene Expression Omnibus (accession number GSE291471; https://www.ncbi.nlm.nih.gov/geo/query/acc.cgi?acc=GSE291471). Original data are available on request from the corresponding author, Aastha Mishra (aastha.mishra@igib.in).

## Abbreviations

AHSP: Alpha hemoglobin stabilizing protein
ALAS2: Aminolevulinate synthase 2
ASL: Above sea level
BCL2L1: BCL2 Like 1
BPs: Biological processes
CBC: Complete blood count
CCL11: C-C motif chemokine ligand 11 or Eotaxin
CCs: Cellular components
CXCL11: C-X-C motif chemokine 11
DEG: Differentially expressed genes
EPB42: Erythrocyte membrane protein band 4.2
FECH: Ferrochelatase
FLT3LG: Fms-related tyrosine kinase 3 ligand
FOXO3: Forkhead box O3
GATA1: GATA binding protein 1
GO: Gene ontology
GOI: Gene of interest
GS: Gene significance
GSEA: Gene set enrichment analysis
GYPA: Glycophorin A
GYPB: Glycophorin B
HA: High altitude
HAPE: High-altitude pulmonary edema
HCT: Hematocrit
HGB: Hemoglobin
HGF: Hepatocyte growth factor
HKG: Housekeeping gene
HR: Heart rate
IL6: Interleukin 6
IL7: Interleukin 7
IL15: Interleukin 15
IL18R1: Interleukin 18 receptor 1
IL1β: Interleukin 1 beta
IMG: Immature granulocytes
IMG: Immature granulocytes
ITGA2B: Integrin alpha IIb
KEGG: Kyoto encyclopedia of genes and genomes
KLF1: Krüppel like factor 1
LINC01093: Long Intergenic Non-Protein Coding RNA 1093
LTA: Lymphotoxin-alpha
Log₂FC: Log₂ fold change
MAP: Mean arterial pressure
MFs: Molecular functions
MM: Module membership
MMP9: Matrix metalloproteinase 9
Mon: Monocytes
MsigDB: Molecular signatures database
NPX: Normalized Protein Expression
Neu: Neutrophils
OLR1: Oxidized low-density lipoprotein receptor 1
OSM: Oncostatin M
PCA: Principal component analysis
PCT: Plateletcrit
PH: Pulmonary hypertension
PHC2: Polyhomeotic homolog 2
P-LCC: Platelet large cell ratio
PLT: Platelets
PR: Pulse rate
RIN: RNA integrity number
ROC: Receiver operating curve
RR: Respiratory rate
RT PCR: Real time PCR
SBP: Systolic blood pressure
SIPA1L2: Signal-induced proliferation-associated 1 like 2
SLC4A1: Solute carrier family 4 anion exchanger member 11
SMAP2: Small ArfGAP2
SMAP2: Small ArfGAP2
SNCA: Synuclein alpha
SNMH: Sonam Norboo Memorial Hospital
SPTA1: Spectrin alpha, erythrocytic 1
SpO₂: Peripheral oxygen saturation
TFRC: Transferrin receptor
TGFA: Pro transforming growth factor alpha
TLR2: Toll-like receptor 2
TLR5: Toll-like receptor 5
TNFSF10: Tumor necrosis factor ligand superfamily member 10
TOM: Topological overlap matrix
UBB: Ubiquitin B
VEGFA: Vascular endothelial growth factor A
WGCNA: Weighted gene co-expression network analysis
ncRNAs: Noncoding RNAs
