# Supplementary material for: Integrative blood profiling uncovers inflammatory network signatures in high-altitude pulmonary edema
Source: Biosci Rep. 2025 Dec 12;45(12):BSR20253746. doi: 10.1042/BSR20253746 (PMC12780694; doi:10.1042/BSR20253746)
Supplement: online supplementary material 1. [file bsr-45-12-BSR20253746-s001.docx]

**Supplementary Figures**


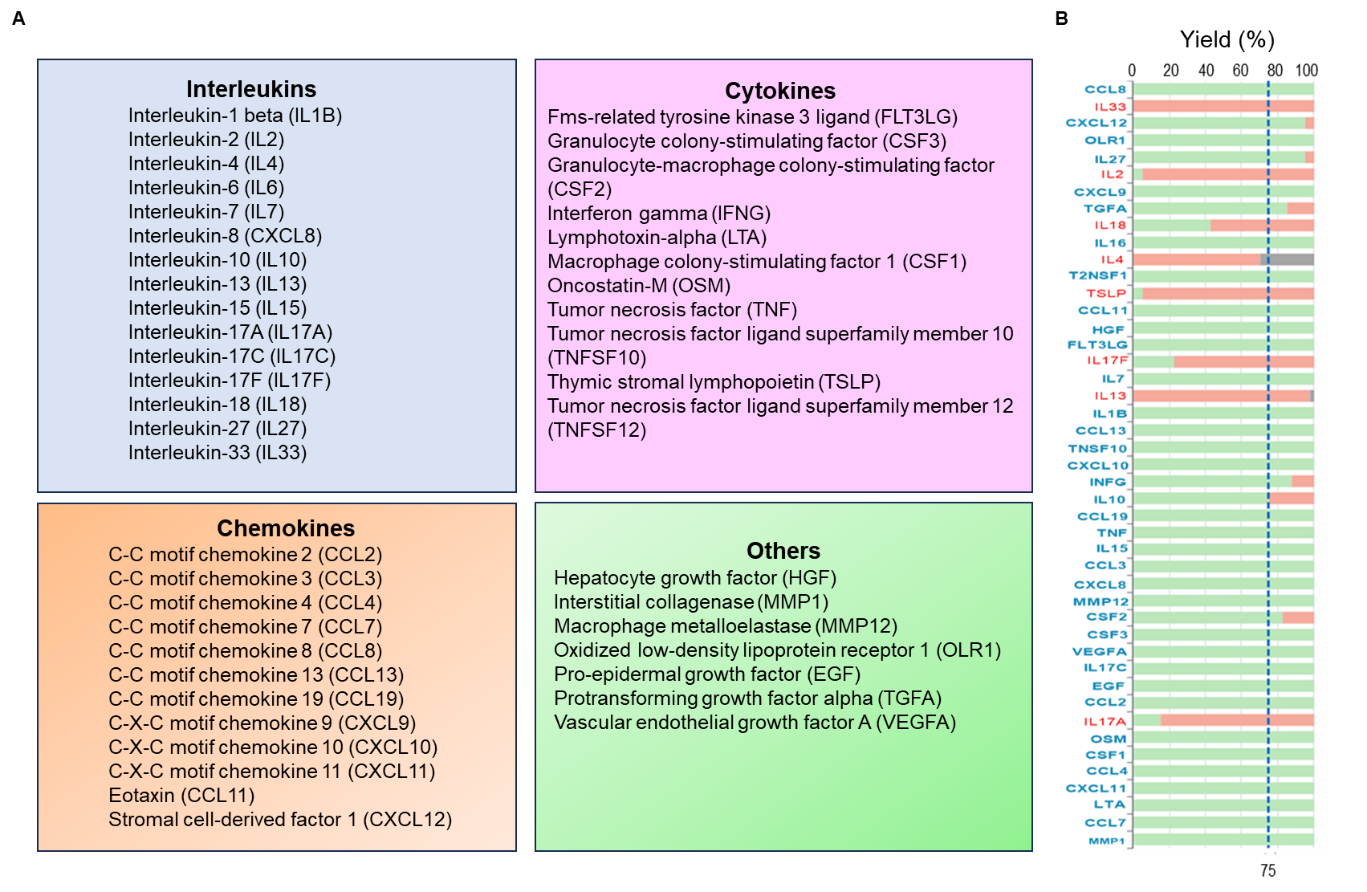


**Figure S1. Targeted plasma cytokine profiles.** (**A**) Segregation of the 45 cytokines into different categories. (**B**) The grouped bar plot represents the % yield of 45 proteins of the cytokine panel, and the red colour highlights the proteins with less than 75% yield.


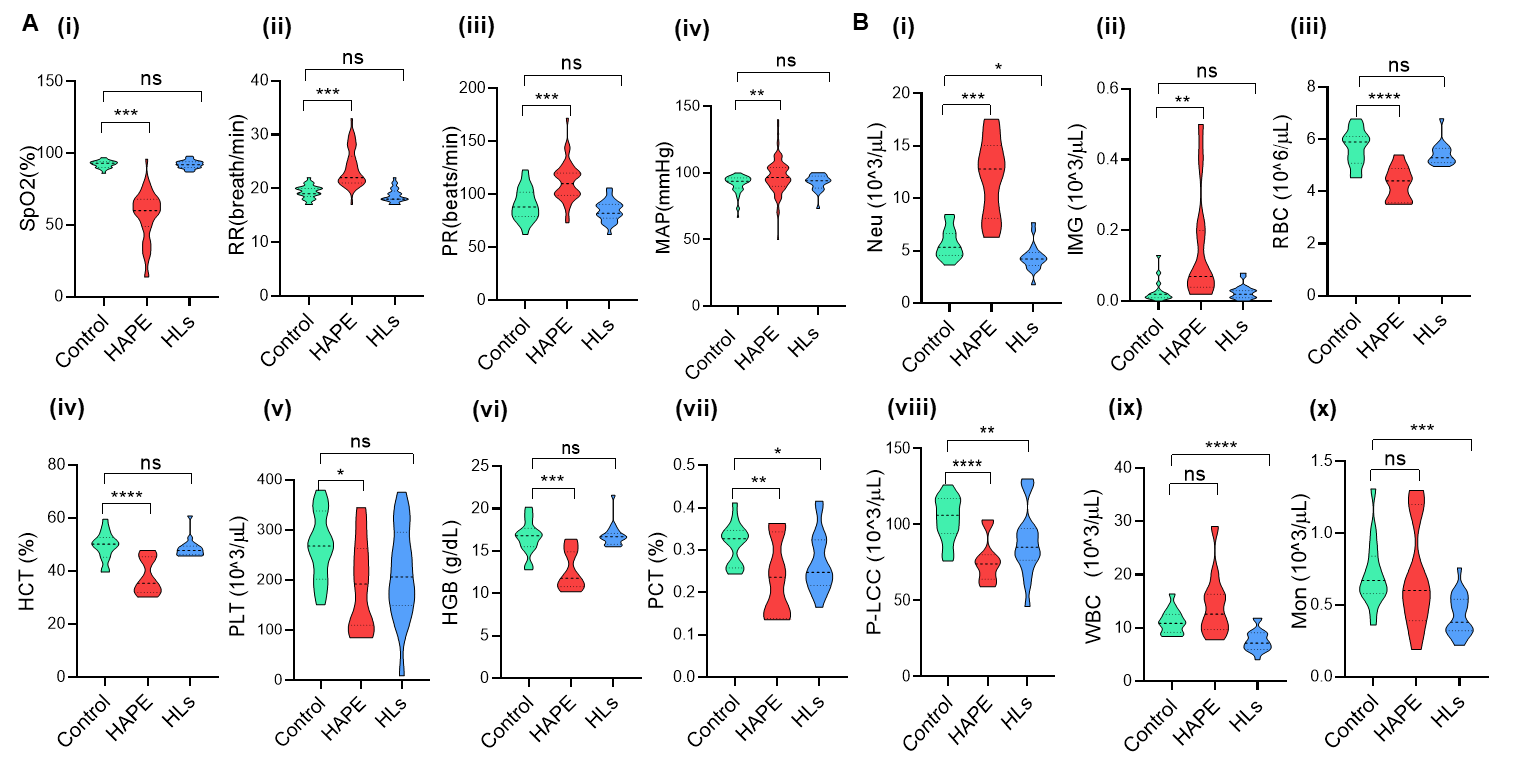


**Figure S2. The clinical profile of the study groups.** (**A**) Violin plot representing the median value of clinical parameters (i) SpO2 (%), (ii) RR (breaths/min), (iii) PR (beats/min), (iv) MAP (mmHg) in controls (n=25), HAPE patients (n=45) and HLs (n=25). (**B**) CBC profile representing (i) Neu(10^3^/µl), (ii) IMG (10^3^/µl), (iii) RBC (10^6^/µl), (iv) HCT (%), (v) PLT (10^3^/µl), (vi) HGB (g/dl), (vii) PCT (%), (viii) P-LCC (10^3^/µl), (ix)WBC (10^3^/µl) and (x) Mon (10^3^/µl) in controls (n=15), HAPE patients (n=15) and HLs (n=15). Here **P < 0.05, **P < 0.01, ***P < 0.001, ****P < 0.0001* and ns is non-significant.


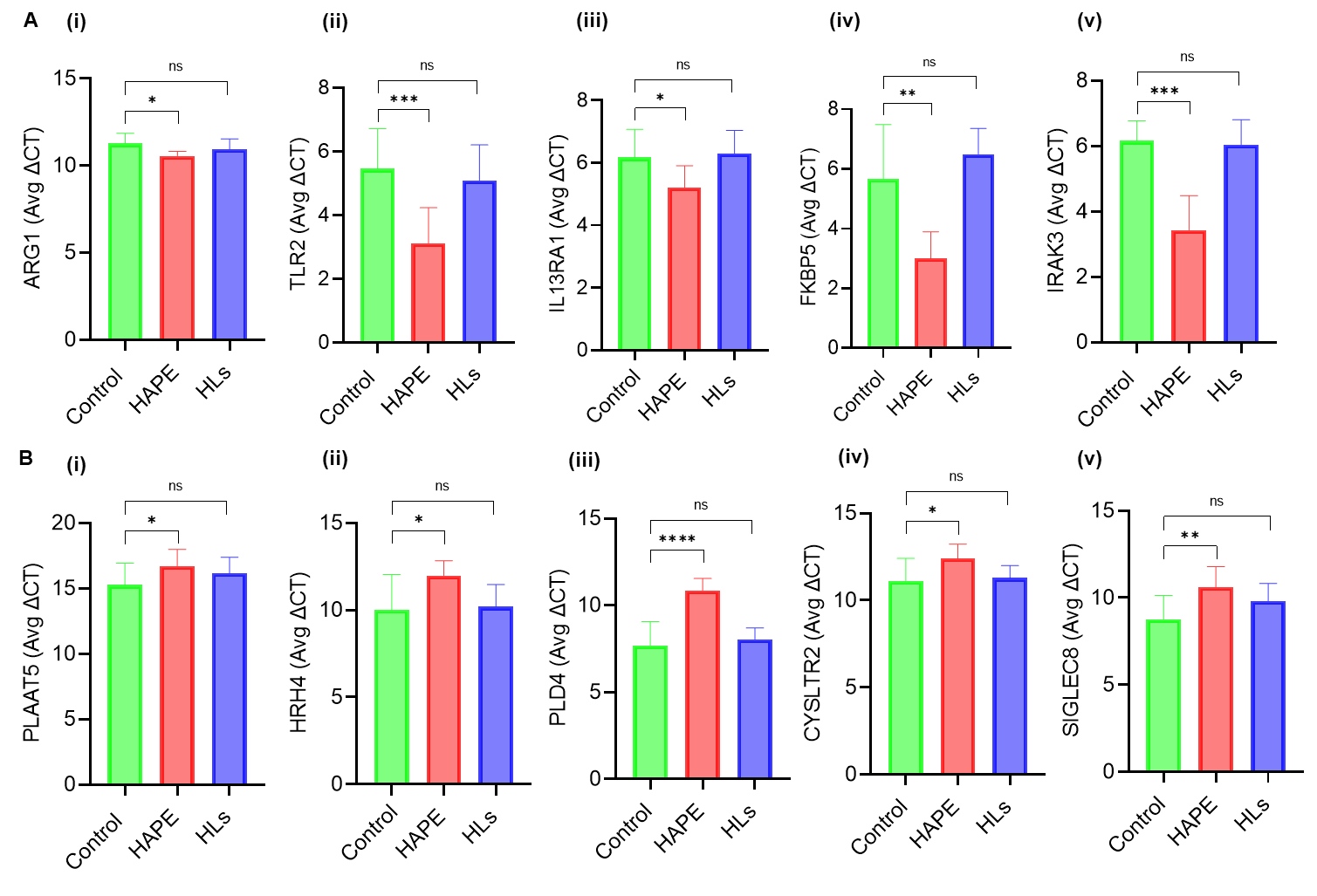


**Figure S3. RT PCR validation of the DEGs in the study groups.** (**A**i-v) The bar plots are representative of the average ΔCT of top 5 upregulated genes. (**B**i-v) The bar plots representative of the average ΔCT of top 5 downregulated genes. The bar represents the mean ΔCT and the error bars represent the SD in controls (n=10), HAPE patients (n=10) and HLs (n=10). Here **P < 0.05, **P < 0.01, ***P < 0.001, ****P < 0.0001* and ns is non-significant.


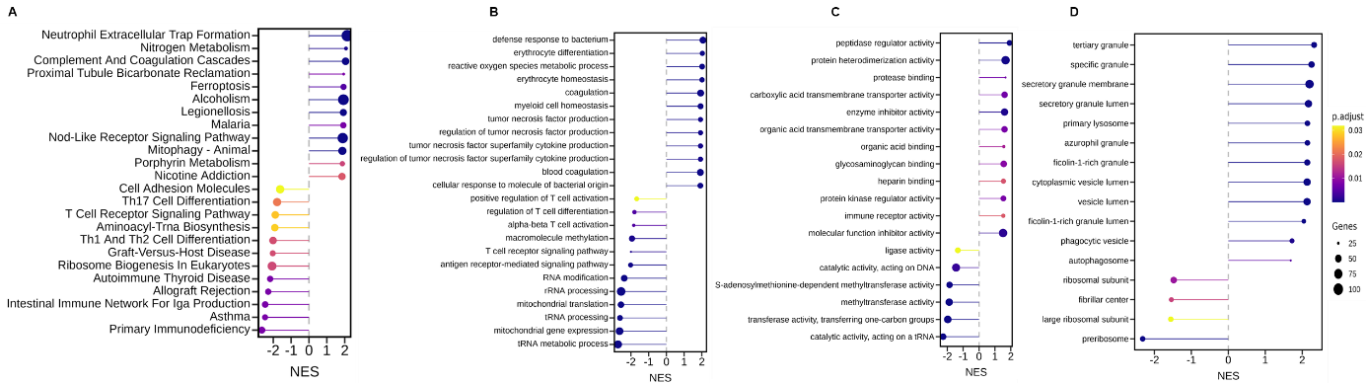


**Figure S4. Pathway enrichment analysis** **of DEGs by KEGG and GO in HAPE patients compared to controls.** (**A**) KEGG pathway, (**B**) BP, (**C**) CC and (**D**)MF. Each stick represents the enrichment level of a specific pathway, with the length of the bar indicating the enrichment score. The depth of the colour represents the log of the *P*- value, and the size of the ball represents the number of genes involved in the pathway.


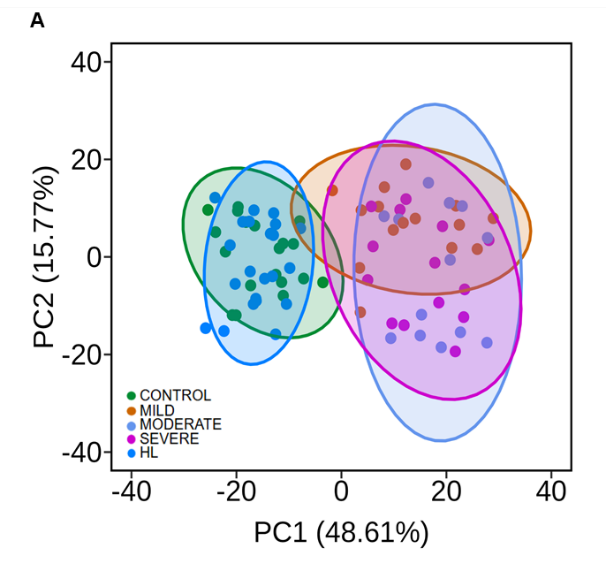


**Figure S5. Clustering in the HAPE severity groups. (A)** PCA represents five separate clusters for control, HLs, and HAPE, along with the three severity groups based on the expression profile.


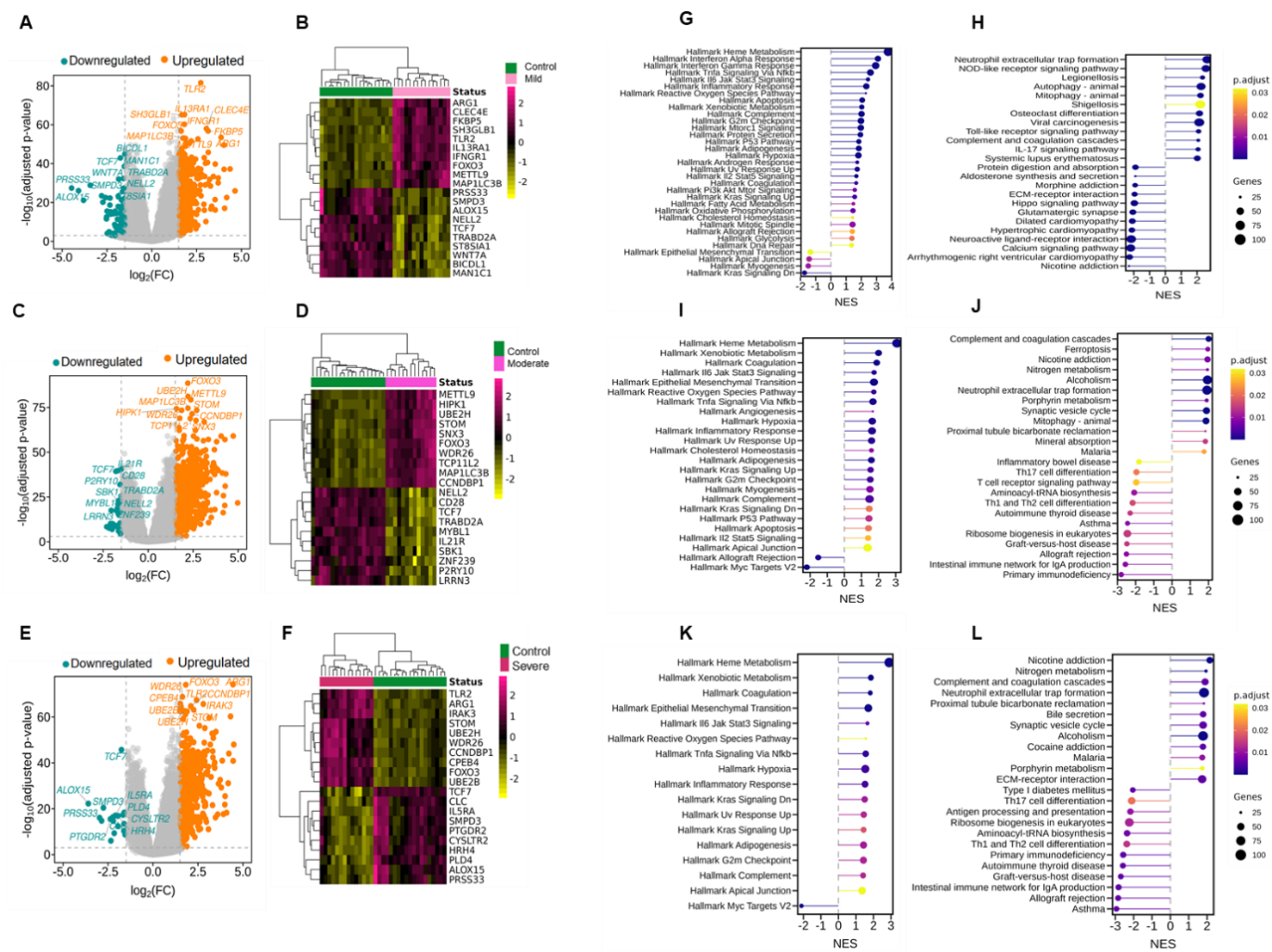


**Figure S6. Identification of DEGs and functional enrichment analysis in HAPE severity groups**. Each point in the volcano plot represents a gene, with the *x*-axis showing the log_2_FC to indicate the magnitude of gene expression changes and the *y*-axis showing the negative log_10_ transformation of the adjusted *P*-value. The orange dots indicate upregulated genes, the green dots indicate downregulated genes, and the grey dots indicate nonsignificant genes in (**A**) Mild vs control, (**C**) Moderate vs control, and (**E**) Severe vs control. Heatmap illustrates patterns of gene expression of the top 10 upregulated and downregulated genes, with the colour scale representing the intensity of gene expression in (**B**) Mild HAPE patients vs. control, (**D**) Moderate HAPE patients vs. control, and (**F**) Severe HAPE patients vs. control. Pathway enrichment analysis of DEGs by msigDB hallmark pathways in (**G**) Mild HAPE patient’s vs control, (**I**) Moderate HAPE patient’s vs control, (**K**) Severe HAPE patient’s vs control, and KEGG analysis in (**H**) Mild HAPE patient’s vs control, (**J**) Moderate HAPE patient’s vs control, (**L**) Severe HAPE patient’s vs control. Each stick represents the enrichment level of a specific pathway, with the length of the bar indicating the enrichment score. The depth of the colour represents the log of the *P-*value, and the size of the ball represents the number of genes involved in the pathway.


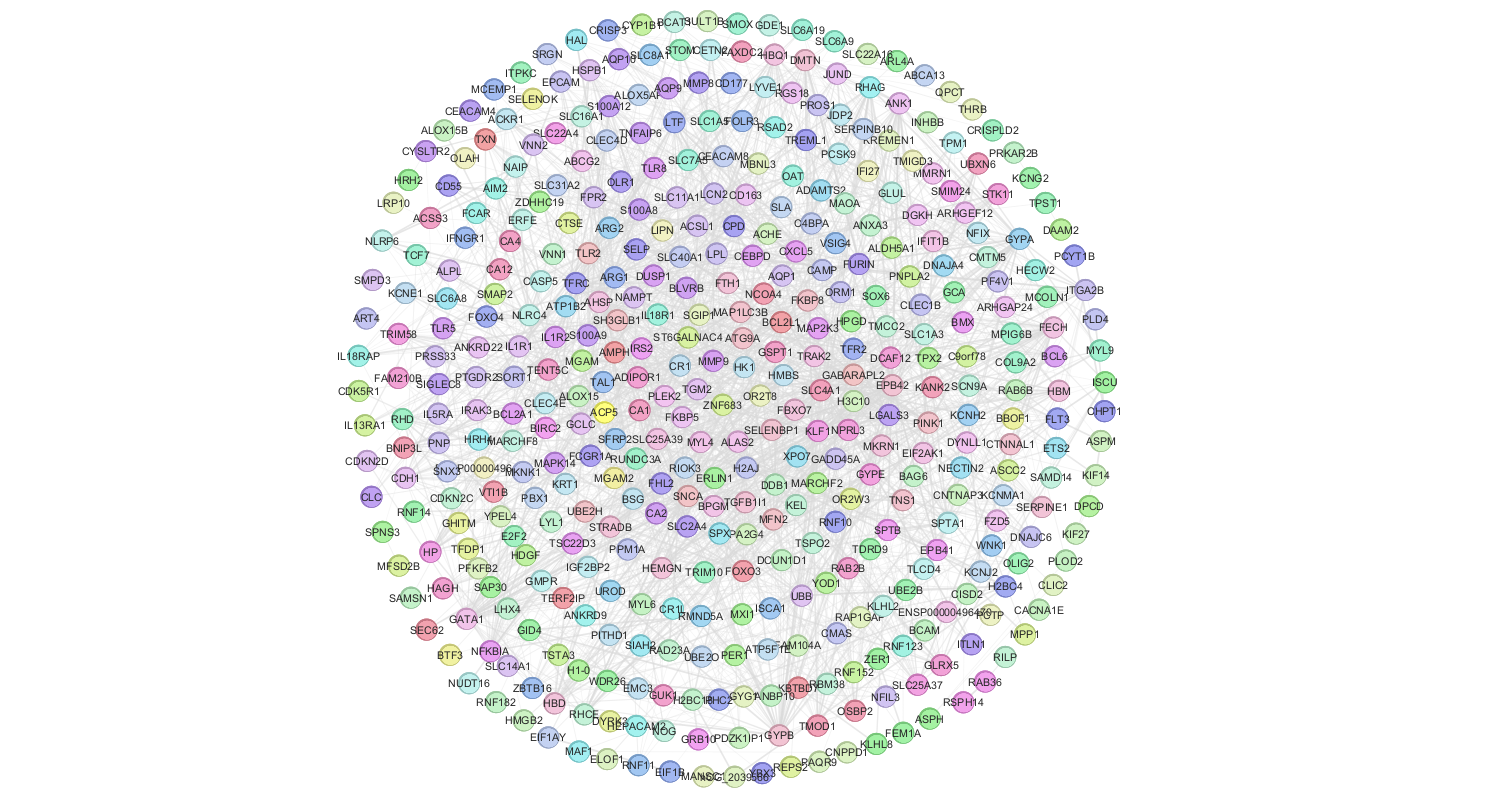


**Figure S7.** The PPI network diagram for the 515 significant DEGs in HAPE patients compared to controls.


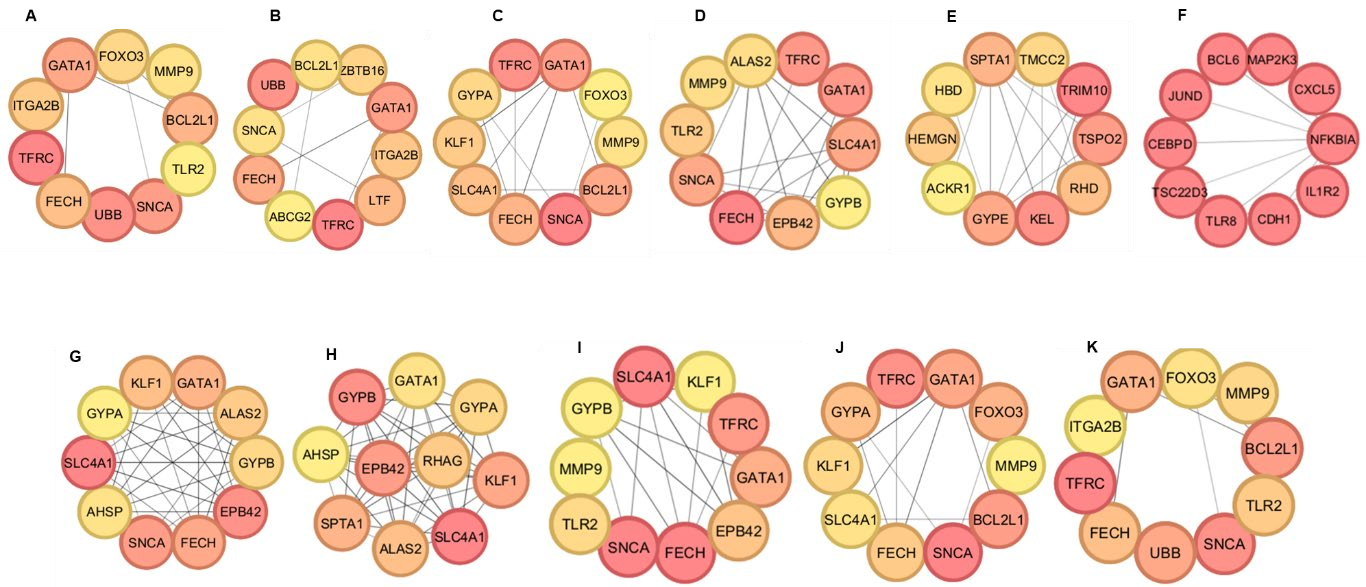


**Figure S8. The PPI network of eleven algorithms of cytohubba for hub gene analysis.** (**A**) Betweenness, (**B**) Bottleneck, (**C**) Closeness, (**D**) Degree, (**E**) DMNC, (**F**) Eccentricity, (**G**) EPC, (**H**) MCC, (**I**) MNC, (**J**) Radiality, (**K**) Stress.


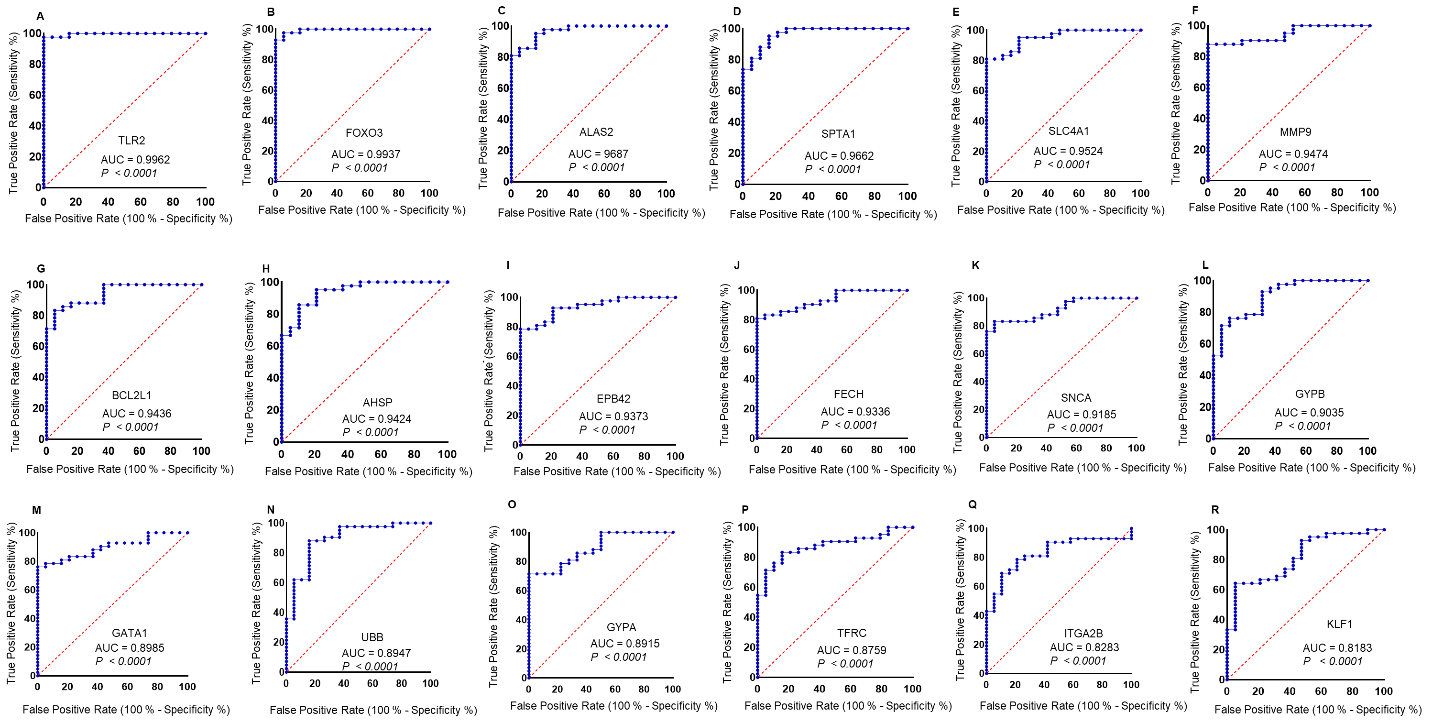


**Figure S9. ROC analysis of the hub genes**. The ROC plot representing the AUC of (**A**) TLR2, (**B**)FOXO3, (**C**)ALAS2, (**D**)SPTA1, (**E**)SLC4A1, (**F**)MMP9, (**G**)BCL2L1, (**H**)AHSP, (**I**)EPB42, (**J**)FECH, (**K**)SNCA, (**L**)GYPB, (**M**)GATA1, (**N**)UBB, (**O**)GYPA, (**P**)TFRC, (**Q**)ITGA2B, (**R**)KLF1.


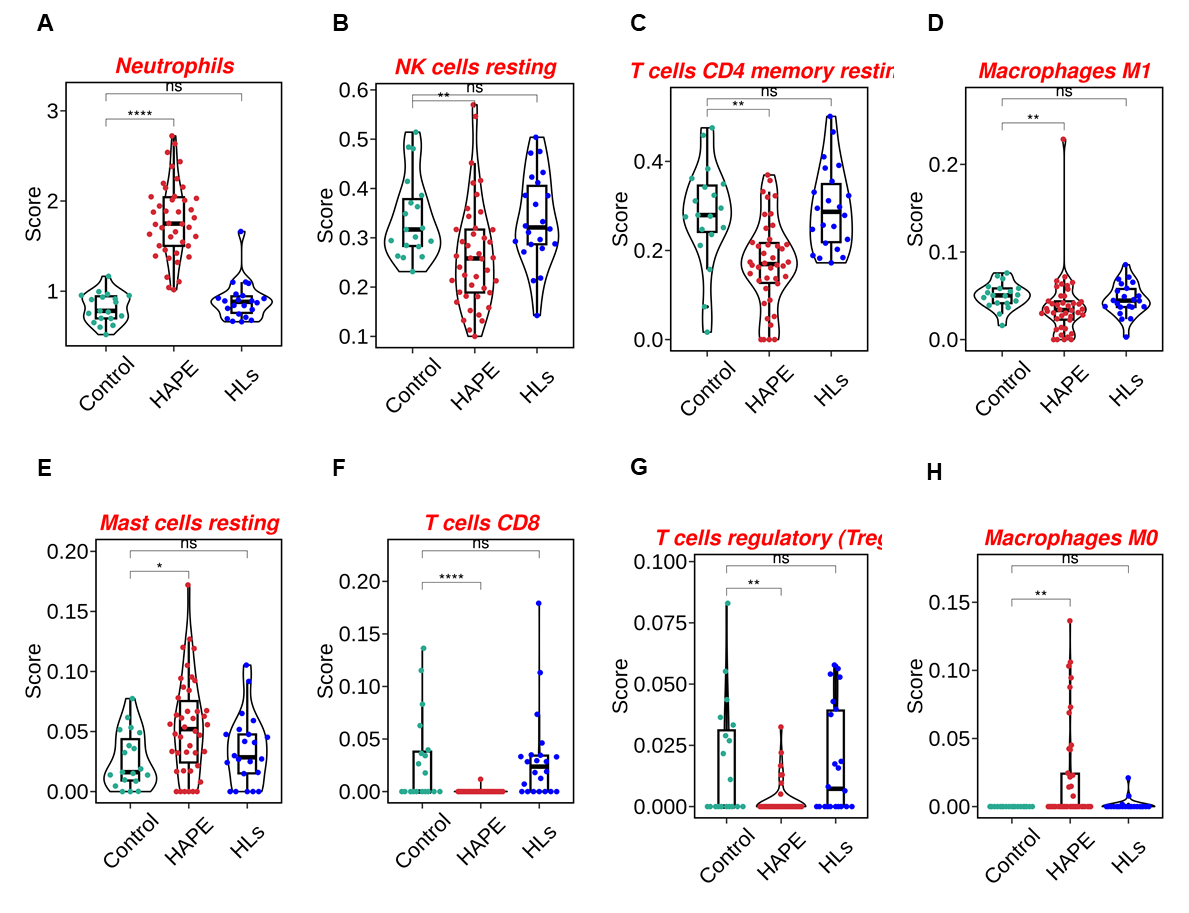


**Figure S10. The beeswarm plot represents the median immune cell abundance levels in the three study groups.** (**A**) Neutrophils, (**B**) NK cells resting, (**C**) T cells CD4 memory resting, (**D**) Macrophage M1, (**E**) Mast cells resting, (**F**) T cells CD8, (**G**) T cells regulatory, (**H**) Macrophages M0. The dots in the beeswarm plots represent the number of samples, and here, **P < 0.05, **P < 0.01, ***P < 0.001, ****P < 0.0001*, and ns is nonsignificant.


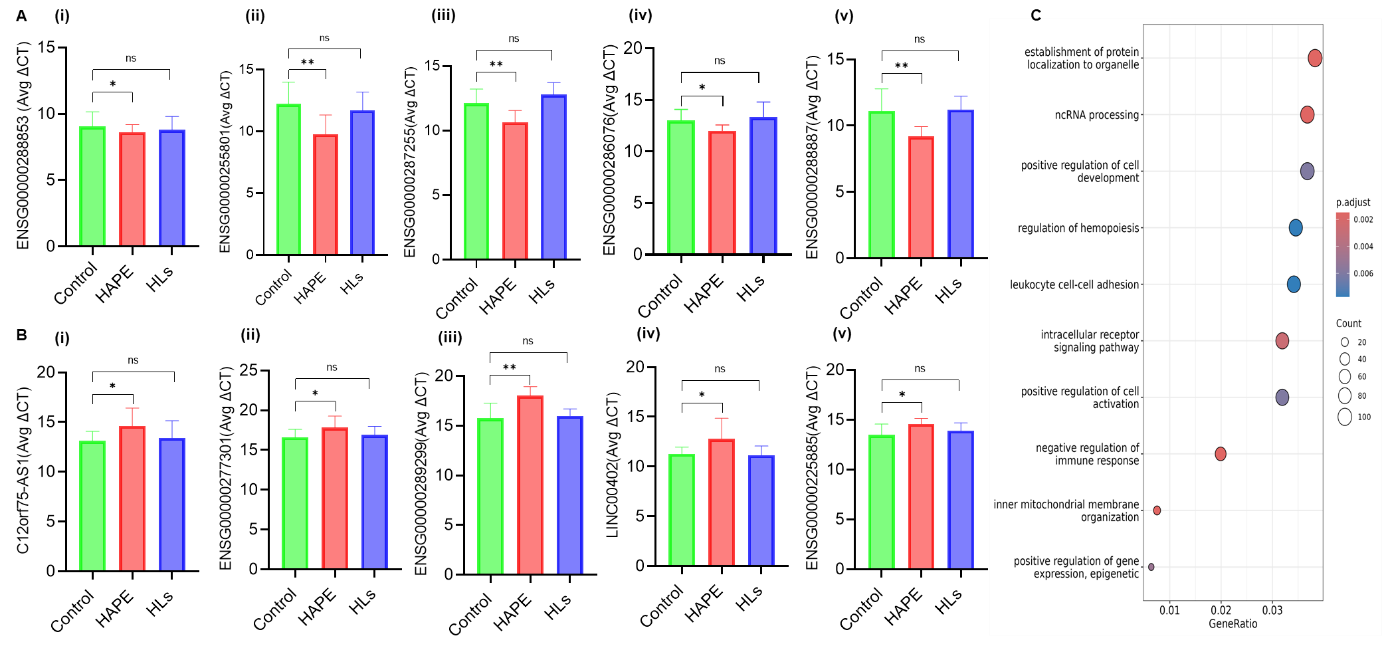


**Figure S11. RT PCR validation of the DE lncRNA in the study groups.**

(**A**i-v) The bar plots representative of the average ΔCT of 5 upregulated LncRNAs. (**B**i-v) The bar plots representative of the average ΔCT of 5 downregulated LncRNAs. The bar represents the mean ΔCT and the error bars represent the SD in controls (n=10), HAPE patients (n=10) and HLs (n=10). (**C**) Overrepresentation analysis of the grey60 module carried out using the enrichGO function of the clusterProfiler. Here **P < 0.05, **P < 0.01, ***P < 0.001, ****P < 0.0001* and ns is non-significant.
