# Supplementary material for: Integrative blood profiling uncovers inflammatory network signatures in high-altitude pulmonary edema
Source: Biosci Rep. 2025 Dec 12;45(12):BSR20253746. doi: 10.1042/BSR20253746 (PMC12780694; doi:10.1042/BSR20253746)
Supplement: online supplementary material 2. [file bsr-45-12-BSR20253746-s002.docx]

**Supplementary Tables**

**Table S1. Real time PCR primers of the genes and their amplified product size**

| **Genes** | **Primer sequence forward** | **Primer sequence reverse** | **Product size**  **(bp)** |
| --- | --- | --- | --- |
| ARG1 | 5’ - ACGGAAGAATCAGCCTGGTG 3’ | 5’ -GTCCACGTCTCTCAAGCCAA 3’ | 281 |
| TLR2 | 5’ - GTAGTTGTGGGTTGAAGCAC 3’ | 5’ -TAATTCCATTGGATGTCAGCAC 3’ | 291 |
| 1L13RA1 | 5’ - GATTACAGAAACTCAGCCAC 3’ | 5’ -CAGACAAATCCTCTCATTCAG 3’ | 208 |
| FKBP5 | 5’ - GCAACAGTAGAAATCCACCTG 3’ | 5’ -AAATACATTGTTCTTCCCGCTG 3’ | 154 |
| IRAK3 | 5’ - CAAACTATGGAGCAGTGTTGAG 3’ | 5’ -TCTCCTTCTCCAATTAGGAAGTC 3’ | 217 |
| PLAAT5 | 5’ - CCCACTCAGGTCTAAATAGCA 3’ | 5’ -CTCAATCAGGTCTCCAGGTC 3’ | 272 |
| HRH4 | 5’ - TTCTTTGTGGGTGTGATCTC 3’ | 5’ -TTCATCCTTCCAAGACTCTG 3’ | 297 |
| PLD4 | 5’ - GAGTTCATCTATGCCTCCGT 3’ | 5’ -GAAGACTTTCACGTCCACAG 3’ | 237 |
| CYSLTR2 | 5’ - AGAGACCTTCAGTCTGAACC 3’ | 5’ -TCTGATACGGAGATGGATGG 3’ | 301 |
| SIGLEC8 | 5’ - ATCTTCATCATAGTGAGGTCCTG 3’ | 5’ -ATCTTGATCTCCGAGTATTCACTG 3’ | 287 |
| ENSG00000288853 | 5’ - GTCCATACATGAACAGCCAC 3’ | 5’ -AAACATAACACAGACCACACC 3’ | 300 |
| ENSG00000255801 | 5’ - ACACAAAGATCAACACCTCC 3’ | 5’ -GTAATCACCACTTCCTCATACC 3’ | 167 |
| ENSG00000287255 | 5’ - GGCAAACAGAAGGACAAAGG 3’ | 5’ -CTTTCCTACCTCACTGGAGTG 3’ | 291 |
| ENSG00000286076 | 5’ - TCTCTTAACACTGTCCTGTAGC 3’ | 5’ -AAGGAAAGCTAATCCTCCTGG 3’ | 226 |
| ENSG00000288887 | 5’ - CTTGAGCCTTGTGAAGAGTG 3’ | 5’ -ACACCAGATATGGTTCTTCCTC 3’ | 188 |
| C12orf75-AS1 | 5’ - GATCACACCACTTAGCTGTCATTTT 3’ | 5’ -TCACTCATACACCTCTTCTCTCT 3’ | 174 |
| ENSG00000277301 | 5’ - CAAGCTGAAGATAACCAGATAAGG 3’ | 5’ -CTGTGGAAGTTGCTGAAGTC 3’ | 280 |
| ENSG00000289299 | 5’ - CCACAAACACAGAATGAATGAG 3’ | 5’ -CAGCATATACACCTATGTTGGA 3’ | 157 |
| LINC00402 | 5’ - TTCTCCCTCCAAATAGCCTG 3’ | 5’ -CACGGAATAACAATCTGAAGATGG 3’ | 244 |
| ENSG00000225885 | 5’ - TCAACTTCTTTCATTCGCCT 3’ | 5’ -ATGAGAACTGTGAACTGAGGA 3’ | 123 |
| OSM | 5’ - GCAGCTGACAAGGTCTGG 3’ | 5’ -CCTGCAGTGCTCTCTCAGTTT 3’ | 203 |
| IL1B | 5’ - CAGATGAAGTGCTCCTTCCA 3’ | 5’ -CACATAAGCCTCGTTATCCCA 3’ | 252 |
| MMP9 | 5’ - TTCACTTTCCTGGGTAAGGAG 3’ | 5’ -CAAACTGTATCCTTGGTCCG 3’ | 135 |

Table represent the RT PCR forward and reverse primer sequences, and the expected PCR product size (in base pairs). The primers were designed to specifically amplify the target gene regions, ensuring accurate and efficient amplification.

**Table S2. Differentially expressed mRNAs between HAPE patient’s vs controls**

| **Genes** | **log2FC** | **Adjusted**  **P-Value** | **Expression type** |
| --- | --- | --- | --- |
| OLAH | 5.109734148 | 1.53E-20 | up |
| RAP1GAP | 4.555778889 | 6.86E-20 | up |
| CA1 | 4.524170596 | 3.27E-30 | up |
| DAAM2 | 4.381225356 | 1.02E-25 | up |
| IFIT1B | 4.339096931 | 3.08E-38 | up |
| FAM83A | 4.337067322 | 5.05E-13 | up |
| ARG1 | 4.219364599 | 6.24E-42 | up |
| CD177 | 4.138498545 | 2.85E-15 | up |
| HBD | 4.070297139 | 2.14E-26 | up |
| MAOA | 3.970110697 | 9.83E-18 | up |
| ERFE | 3.744314603 | 2.29E-10 | up |
| AHSP | 3.72676422 | 2.75E-24 | up |
| RNF182 | 3.687365648 | 3.30E-14 | up |
| VSIG4 | 3.621790991 | 5.33E-19 | up |
| ECHDC3 | 3.528558153 | 9.36E-27 | up |
| BPGM | 3.524591066 | 5.32E-33 | up |
| GYPA | 3.49533689 | 9.40E-18 | up |
| HEPACAM2 | 3.482461917 | 5.43E-23 | up |
| IFI27 | 3.410575335 | 1.73E-10 | up |
| CA2 | 3.394911522 | 1.35E-24 | up |
| SLFN14 | 3.287649687 | 8.24E-31 | up |
| TMCC2 | 3.274023791 | 3.73E-30 | up |
| SLC1A3 | 3.225596008 | 9.89E-20 | up |
| ARL4A | 3.222166054 | 6.60E-26 | up |
| HES6 | 3.220656302 | 7.27E-09 | up |
| ART4 | 3.215059717 | 3.73E-11 | up |
| IL1R2 | 3.214205267 | 6.03E-33 | up |
| MCEMP1 | 3.212430251 | 5.99E-21 | up |
| PFKFB2 | 3.202379811 | 5.53E-30 | up |
| DYRK3 | 3.164462941 | 2.28E-23 | up |
| FKBP5 | 3.147934191 | 3.16E-39 | up |
| ALAS2 | 3.103795425 | 1.47E-22 | up |
| SCRG1 | 3.068279897 | 6.05E-11 | up |
| OSBP2 | 3.013928551 | 1.65E-22 | up |
| IL18R1 | 2.991707163 | 1.26E-23 | up |
| CLEC4E | 2.984011458 | 3.03E-38 | up |
| ANKRD9 | 2.933454588 | 2.92E-21 | up |
| KCNE1 | 2.881662905 | 1.00E-28 | up |
| YOD1 | 2.86364838 | 8.48E-26 | up |
| AMPH | 2.85917098 | 3.23E-14 | up |
| GFUS | 2.846597989 | 3.84E-18 | up |
| GYPB | 2.841071065 | 7.71E-17 | up |
| MMP8 | 2.801441002 | 7.11E-13 | up |
| ZNF608 | 2.792244909 | 1.79E-26 | up |
| CLIC2 | 2.787649377 | 1.43E-25 | up |
| SPTB | 2.780695431 | 2.65E-21 | up |
| ACHE | 2.776043236 | 1.04E-13 | up |
| RIOK3 | 2.763163376 | 7.52E-28 | up |
| IL18RAP | 2.751620639 | 1.69E-22 | up |
| KRT1 | 2.751485932 | 3.05E-12 | up |
| GRB10 | 2.741528074 | 2.61E-28 | up |
| OR2T8 | 2.738828588 | 2.99E-08 | up |
| FAM210B | 2.73836984 | 1.77E-20 | up |
| SPTA1 | 2.738075355 | 1.49E-22 | up |
| IRAK3 | 2.684922508 | 4.94E-39 | up |
| GADD45A | 2.684249624 | 3.90E-26 | up |
| EIF1AY | 2.681791223 | 3.04E-21 | up |
| HBM | 2.677850168 | 5.36E-17 | up |
| TPST1 | 2.671887428 | 1.01E-24 | up |
| VNN1 | 2.670344422 | 1.13E-20 | up |
| SELENBP1 | 2.668267797 | 2.36E-19 | up |
| PAQR9 | 2.660281908 | 1.18E-10 | up |
| ENSG00000269711 | 2.658157603 | 4.94E-07 | up |
| PAGE2B | 2.646998531 | 3.62E-10 | up |
| TRIM58 | 2.643217024 | 3.86E-22 | up |
| NCOA4 | 2.639949504 | 4.05E-34 | up |
| TENT5C | 2.631136977 | 3.48E-23 | up |
| RHAG | 2.608932125 | 7.81E-12 | up |
| PLSCR4 | 2.602481873 | 5.36E-17 | up |
| KCNMA1 | 2.600198671 | 1.80E-05 | up |
| EPB42 | 2.598122913 | 2.12E-18 | up |
| SIAH2 | 2.594481327 | 2.12E-24 | up |
| ANK1 | 2.567916566 | 6.70E-23 | up |
| CTNNAL1 | 2.56258904 | 8.00E-18 | up |
| S100A12 | 2.559510931 | 1.15E-21 | up |
| GMPR | 2.556452537 | 1.74E-19 | up |
| RUNDC3A | 2.554492659 | 3.60E-17 | up |
| TSPAN7 | 2.551086768 | 1.73E-08 | up |
| MXI1 | 2.546944406 | 9.59E-28 | up |
| FHDC1 | 2.540366887 | 7.24E-23 | up |
| ARG2 | 2.53287669 | 3.81E-08 | up |
| FECH | 2.527447972 | 2.67E-20 | up |
| ANKRD22 | 2.525232837 | 3.73E-13 | up |
| TLR2 | 2.517274855 | 6.81E-45 | up |
| GLRX5 | 2.512755148 | 1.26E-19 | up |
| PBX1 | 2.511632591 | 2.55E-22 | up |
| CMBL | 2.507950915 | 1.17E-07 | up |
| FLT3 | 2.493512904 | 5.49E-25 | up |
| ARHGEF12 | 2.4870265 | 1.63E-26 | up |
| KANK2 | 2.477058523 | 5.90E-19 | up |
| IL1R1 | 2.475617405 | 2.55E-22 | up |
| CISD2 | 2.464815059 | 1.41E-23 | up |
| ASPH | 2.452212771 | 4.69E-26 | up |
| ALOX15B | 2.442187378 | 5.01E-08 | up |
| CLEC4D | 2.440242635 | 1.31E-22 | up |
| ZBTB16 | 2.440131187 | 9.82E-14 | up |
| MACIR | 2.437055372 | 1.55E-19 | up |
| IGF2BP2 | 2.428753325 | 2.42E-21 | up |
| HP | 2.413347225 | 6.25E-12 | up |
| INHBB | 2.410920318 | 1.63E-09 | up |
| SLC4A1 | 2.406722669 | 1.39E-17 | up |
| SLC6A19 | 2.386172133 | 0.000112858 | up |
| PNP | 2.38590476 | 1.01E-23 | up |
| ADAMTS2 | 2.379325507 | 2.18E-06 | up |
| TSPO2 | 2.378051711 | 8.02E-09 | up |
| PER1 | 2.370601948 | 1.49E-19 | up |
| ORM1 | 2.35854676 | 3.44E-08 | up |
| RHD | 2.351620907 | 7.76E-14 | up |
| MPP1 | 2.348980459 | 1.32E-23 | up |
| TLCD4 | 2.347828313 | 1.06E-09 | up |
| UBE2O | 2.345772802 | 9.30E-25 | up |
| SLC2A4 | 2.342575098 | 4.57E-07 | up |
| BMX | 2.341377328 | 3.68E-15 | up |
| SRGAP1 | 2.33934004 | 8.28E-11 | up |
| MGAM2 | 2.33450453 | 1.29E-27 | up |
| TMOD1 | 2.327406119 | 2.21E-17 | up |
| SHISA7 | 2.321259795 | 5.18E-10 | up |
| TGM2 | 2.303250433 | 3.80E-14 | up |
| CREG1 | 2.297340033 | 1.50E-26 | up |
| MBNL3 | 2.296622354 | 2.48E-20 | up |
| CA4 | 2.293980471 | 4.28E-16 | up |
| TERF2IP | 2.289543896 | 1.26E-29 | up |
| ALDH5A1 | 2.286349058 | 1.70E-25 | up |
| CD163 | 2.283567153 | 2.87E-15 | up |
| THEM5 | 2.282078612 | 4.95E-07 | up |
| STOM | 2.264215857 | 3.96E-35 | up |
| NSUN7 | 2.260470138 | 1.88E-17 | up |
| BNIP3L | 2.259440844 | 1.78E-24 | up |
| SDK1 | 2.252640828 | 2.69E-07 | up |
| SHROOM4 | 2.249362105 | 7.97E-16 | up |
| BCAM | 2.242721734 | 4.89E-09 | up |
| CDKN2C | 2.240541545 | 5.37E-18 | up |
| HEMGN | 2.235060734 | 1.08E-15 | up |
| TRIM10 | 2.234294757 | 3.02E-14 | up |
| IRS2 | 2.233864189 | 5.20E-24 | up |
| TFDP1 | 2.232738369 | 2.89E-22 | up |
| CD163L1 | 2.229090118 | 8.02E-08 | up |
| PKD1L3 | 2.224804651 | 4.57E-07 | up |
| GSPT1 | 2.220280862 | 2.49E-19 | up |
| SIPA1L2 | 2.207742116 | 9.95E-30 | up |
| EPCAM | 2.2044471 | 1.28E-05 | up |
| SEC62 | 2.199300377 | 5.04E-32 | up |
| SAP30 | 2.198855756 | 1.37E-18 | up |
| GABARAPL2 | 2.198763649 | 2.99E-31 | up |
| S100A8 | 2.194423737 | 6.33E-18 | up |
| SLC26A8 | 2.19367212 | 1.61E-26 | up |
| NUDT16 | 2.189115163 | 1.71E-24 | up |
| NUDT4 | 2.186899722 | 1.42E-23 | up |
| CNTNAP3C | 2.163427081 | 4.23E-14 | up |
| PCSK1N | 2.151921519 | 1.80E-11 | up |
| ANXA3 | 2.150898965 | 4.37E-19 | up |
| SLC25A37 | 2.150812132 | 4.15E-31 | up |
| PCYT1B | 2.149140339 | 1.72E-09 | up |
| SAMSN1 | 2.146257317 | 7.57E-25 | up |
| BCL2A1 | 2.143426615 | 4.66E-21 | up |
| H2BC26 | 2.13880565 | 1.84E-14 | up |
| ZMAT2 | 2.134323851 | 4.26E-23 | up |
| TPM1 | 2.125654264 | 5.91E-19 | up |
| RNF10 | 2.123895174 | 1.53E-25 | up |
| HMGB2 | 2.121530146 | 3.16E-20 | up |
| CCNDBP1 | 2.120981764 | 5.34E-39 | up |
| ABCG2 | 2.11899388 | 3.71E-14 | up |
| ADIPOR1 | 2.117155146 | 7.74E-19 | up |
| NFIX | 2.1161567 | 3.24E-16 | up |
| CRISP3 | 2.115594696 | 1.22E-08 | up |
| ACKR1 | 2.113961484 | 2.63E-10 | up |
| RAB6B | 2.113529664 | 3.78E-10 | up |
| CNTNAP3 | 2.112900479 | 4.61E-14 | up |
| HK1 | 2.10746239 | 9.03E-24 | up |
| PA2G4 | 2.104946784 | 5.45E-16 | up |
| ST6GALNAC3 | 2.104554565 | 4.58E-19 | up |
| C9orf78 | 2.103220502 | 2.12E-13 | up |
| COL9A2 | 2.101329936 | 1.31E-11 | up |
| SLC8A1 | 2.100622575 | 7.67E-23 | up |
| RHCE | 2.099321846 | 9.96E-10 | up |
| AQP1 | 2.098189314 | 1.52E-06 | up |
| PLEK2 | 2.095109488 | 7.15E-11 | up |
| OR2W3 | 2.090569737 | 1.00E-12 | up |
| PF4V1 | 2.082220202 | 8.23E-07 | up |
| SLC1A5 | 2.081876035 | 4.83E-15 | up |
| RAD23A | 2.080365426 | 1.64E-15 | up |
| BCL2L1 | 2.074891415 | 1.17E-18 | up |
| SMIM24 | 2.070384776 | 2.76E-08 | up |
| BBOF1 | 2.069885413 | 5.89E-16 | up |
| LRRN1 | 2.067728675 | 2.25E-18 | up |
| GALNT5 | 2.066439117 | 8.03E-12 | up |
| HBQ1 | 2.065487099 | 1.80E-11 | up |
| STRADB | 2.061778126 | 4.23E-14 | up |
| FZD5 | 2.060571577 | 6.50E-14 | up |
| RAB2B | 2.060169935 | 1.18E-17 | up |
| SLC5A9 | 2.058961241 | 1.07E-10 | up |
| PCSK9 | 2.058594906 | 0.000563264 | up |
| UBB | 2.057976683 | 4.70E-12 | up |
| TRAK2 | 2.055118015 | 1.24E-25 | up |
| TMIGD3 | 2.053398152 | 1.44E-10 | up |
| MGAM | 2.05040308 | 3.19E-24 | up |
| KEL | 2.050358554 | 1.66E-13 | up |
| KIF27 | 2.047882798 | 4.48E-20 | up |
| YPEL4 | 2.04161413 | 1.10E-11 | up |
| C8orf88 | 2.040165778 | 6.21E-20 | up |
| TFR2 | 2.037655377 | 4.95E-11 | up |
| TLR5 | 2.037521929 | 9.36E-27 | up |
| CLEC1B | 2.034473745 | 5.82E-11 | up |
| MARCHF8 | 2.032354503 | 1.34E-24 | up |
| SLC7A5 | 2.029094363 | 1.59E-14 | up |
| KCNJ2 | 2.023233674 | 2.26E-29 | up |
| DCAF12 | 2.022572964 | 4.33E-17 | up |
| RNF11 | 2.017088621 | 4.73E-27 | up |
| TMEM158 | 2.016238208 | 2.48E-05 | up |
| CRISPLD2 | 2.011131758 | 1.62E-23 | up |
| DGKH | 2.010831152 | 4.06E-16 | up |
| OAT | 2.009382942 | 3.55E-18 | up |
| LYVE1 | 2.008119239 | 1.10E-18 | up |
| A3GALT2 | 2.007794753 | 5.09E-15 | up |
| MKRN1 | 2.006355931 | 3.37E-22 | up |
| MANSC1 | 2.006022503 | 2.89E-27 | up |
| MMP9 | 2.003084924 | 2.52E-11 | up |
| SULT1B1 | 1.999576898 | 1.60E-31 | up |
| ARHGEF37 | 1.997145507 | 5.97E-14 | up |
| METTL9 | 1.995442124 | 9.60E-39 | up |
| SOX6 | 1.994652068 | 3.45E-17 | up |
| YBX3 | 1.993802471 | 2.10E-14 | up |
| MAB21L3 | 1.990980704 | 6.49E-14 | up |
| ENSG00000286231 | 1.989000381 | 4.43E-15 | up |
| CNPPD1 | 1.988912156 | 8.65E-21 | up |
| SUCNR1 | 1.985637906 | 7.08E-09 | up |
| FOXO4 | 1.983441922 | 1.06E-16 | up |
| DNAJC6 | 1.977675432 | 3.55E-15 | up |
| ITLN1 | 1.973380515 | 2.85E-06 | up |
| FURIN | 1.972300549 | 2.87E-27 | up |
| MOSPD1 | 1.972272656 | 1.14E-22 | up |
| EPB41 | 1.969409007 | 2.04E-23 | up |
| LIPN | 1.966998705 | 7.17E-15 | up |
| ISCA1 | 1.957869457 | 1.49E-19 | up |
| TNS1 | 1.956197039 | 4.84E-14 | up |
| SNX3 | 1.956083419 | 1.30E-33 | up |
| HECW2 | 1.954787727 | 3.77E-20 | up |
| SELENOK | 1.947392891 | 4.84E-17 | up |
| TDRD9 | 1.946847001 | 1.74E-11 | up |
| RNF14 | 1.946294799 | 2.55E-25 | up |
| EIF2AK1 | 1.94570988 | 5.70E-17 | up |
| CYYR1 | 1.944240781 | 3.71E-08 | up |
| PINK1 | 1.942748866 | 2.18E-22 | up |
| ACSL1 | 1.941786565 | 4.78E-26 | up |
| WNK1 | 1.92368655 | 1.20E-23 | up |
| H1-0 | 1.923632854 | 2.29E-10 | up |
| SORT1 | 1.917401643 | 1.00E-21 | up |
| SLC40A1 | 1.907698374 | 1.69E-31 | up |
| SNCA | 1.906456804 | 2.23E-14 | up |
| UROD | 1.904119764 | 3.83E-15 | up |
| TNFAIP6 | 1.900865157 | 5.78E-13 | up |
| EIF1B | 1.898913913 | 1.20E-17 | up |
| SFRP2 | 1.895515812 | 1.47E-07 | up |
| BLVRB | 1.895455454 | 7.17E-13 | up |
| ATP1B2 | 1.892827507 | 3.27E-09 | up |
| ST13 | 1.892134275 | 8.44E-20 | up |
| MAP1LC3B | 1.888171972 | 1.19E-38 | up |
| HMBS | 1.886237853 | 3.53E-11 | up |
| ISCU | 1.879989104 | 5.21E-21 | up |
| SMIM5 | 1.879036696 | 2.45E-11 | up |
| SLC22A16 | 1.876003471 | 1.88E-16 | up |
| SLC6A8 | 1.875658251 | 8.08E-12 | up |
| GPR146 | 1.874603325 | 6.47E-13 | up |
| LGALS3 | 1.87430126 | 1.36E-13 | up |
| TMEM200B | 1.871783061 | 1.11E-07 | up |
| FOXO3 | 1.868356509 | 1.06E-42 | up |
| SERPINE1 | 1.867899601 | 5.58E-08 | up |
| CTSE | 1.865971386 | 1.56E-09 | up |
| NECTIN2 | 1.864217492 | 4.03E-05 | up |
| PITHD1 | 1.863648022 | 3.27E-13 | up |
| DUSP1 | 1.862584768 | 1.33E-19 | up |
| SLC14A1 | 1.859372533 | 1.67E-16 | up |
| CETN2 | 1.854251357 | 1.53E-15 | up |
| E2F2 | 1.854068932 | 1.24E-15 | up |
| ZDHHC19 | 1.850987545 | 1.21E-05 | up |
| BSG | 1.850390964 | 2.11E-13 | up |
| CHPT1 | 1.844717142 | 1.44E-25 | up |
| C1orf116 | 1.842959225 | 2.79E-08 | up |
| DMTN | 1.841807828 | 5.41E-13 | up |
| GCA | 1.841319498 | 1.66E-25 | up |
| ARHGAP29 | 1.841036138 | 6.00E-08 | up |
| FCAR | 1.834932077 | 4.68E-21 | up |
| CASP5 | 1.830894406 | 1.70E-14 | up |
| SMAP2 | 1.826455965 | 2.40E-30 | up |
| FAM104A | 1.826158913 | 3.00E-20 | up |
| VTI1B | 1.82522351 | 3.97E-17 | up |
| CR1L | 1.825200938 | 2.90E-16 | up |
| GPER1 | 1.82127492 | 4.96E-06 | up |
| CAMP | 1.819443178 | 1.25E-12 | up |
| MYL4 | 1.818502528 | 1.54E-10 | up |
| SMIM1 | 1.81609852 | 6.53E-06 | up |
| TAL1 | 1.813254262 | 1.76E-15 | up |
| PHC2 | 1.81130081 | 2.19E-23 | up |
| REXO2 | 1.809184956 | 1.07E-14 | up |
| PNPLA2 | 1.808862849 | 1.96E-14 | up |
| PROS1 | 1.808659464 | 9.23E-10 | up |
| ERLIN1 | 1.800552993 | 6.82E-18 | up |
| ENSG00000288796 | 1.796079981 | 2.21E-06 | up |
| RNF152 | 1.795738763 | 3.02E-06 | up |
| KCNG2 | 1.795311444 | 4.61E-06 | up |
| ATP5F1E | 1.79478259 | 7.61E-16 | up |
| HDGF | 1.794715205 | 1.39E-13 | up |
| KLHL2 | 1.793320751 | 3.54E-27 | up |
| TXN | 1.79267314 | 4.86E-29 | up |
| FKBP8 | 1.791737433 | 2.69E-13 | up |
| TMEM86B | 1.789831983 | 4.23E-12 | up |
| C4BPA | 1.789335732 | 0.010301177 | up |
| ALOX5AP | 1.785042791 | 1.30E-18 | up |
| STMP1 | 1.774752413 | 1.74E-17 | up |
| LRRC2 | 1.774332545 | 0.000168773 | up |
| MFSD2B | 1.773125093 | 3.36E-12 | up |
| PPM1A | 1.771258074 | 2.92E-35 | up |
| DNAJA4 | 1.770278868 | 1.33E-16 | up |
| CA12 | 1.769919895 | 9.50E-05 | up |
| EMC3 | 1.769326872 | 3.74E-19 | up |
| MAK | 1.767943596 | 1.41E-17 | up |
| SAMD15 | 1.767878376 | 8.95E-07 | up |
| CD55 | 1.766921576 | 7.58E-32 | up |
| UBE2H | 1.766790716 | 4.55E-32 | up |
| RAB36 | 1.766722354 | 5.93E-14 | up |
| CEBPD | 1.761657661 | 1.43E-17 | up |
| HSPB1 | 1.759475328 | 8.85E-10 | up |
| CDK5R1 | 1.758744987 | 1.46E-12 | up |
| MMRN1 | 1.756545092 | 4.83E-10 | up |
| ENSG00000284931 | 1.7560087 | 0.011286441 | up |
| FAT1 | 1.752980922 | 7.35E-05 | up |
| H3C10 | 1.752723268 | 3.70E-13 | up |
| CDKN2D | 1.752659048 | 1.93E-20 | up |
| ZER1 | 1.750604878 | 4.36E-21 | up |
| GALNT14 | 1.749821412 | 1.91E-13 | up |
| MAN2A2 | 1.744286581 | 1.50E-26 | up |
| MYL6 | 1.744132116 | 6.57E-22 | up |
| KLF1 | 1.740932832 | 4.48E-08 | up |
| TGFB1I1 | 1.738228684 | 1.68E-06 | up |
| CR1 | 1.737709629 | 3.44E-19 | up |
| NAIP | 1.732055631 | 3.85E-18 | up |
| TFRC | 1.731907498 | 3.80E-13 | up |
| HPGD | 1.731489634 | 2.70E-07 | up |
| MKNK1 | 1.730217428 | 2.66E-24 | up |
| SGIP1 | 1.728425764 | 1.49E-05 | up |
| SCN9A | 1.728033724 | 1.13E-17 | up |
| RGS18 | 1.727595996 | 2.01E-28 | up |
| ABCA13 | 1.727548848 | 3.13E-12 | up |
| IL13RA1 | 1.725984471 | 1.54E-39 | up |
| TMEM63B | 1.717995791 | 2.08E-12 | up |
| C10orf105 | 1.716668368 | 7.82E-22 | up |
| CEACAM8 | 1.713865225 | 1.88E-07 | up |
| PHF24 | 1.711973961 | 1.13E-06 | up |
| SRGN | 1.711225465 | 1.85E-29 | up |
| SLC6A9 | 1.710249122 | 2.19E-07 | up |
| SPATA6 | 1.710043405 | 2.52E-15 | up |
| REPS2 | 1.709331065 | 9.84E-25 | up |
| SVBP | 1.707214735 | 4.70E-12 | up |
| DPCD | 1.703471321 | 5.73E-11 | up |
| FHL2 | 1.702746007 | 3.93E-07 | up |
| KREMEN1 | 1.700694339 | 6.92E-13 | up |
| PCTP | 1.697625226 | 2.07E-32 | up |
| RSAD2 | 1.694317455 | NA | ns |
| DCUN1D1 | 1.693739723 | 1.75E-32 | up |
| GID4 | 1.690668626 | 1.50E-17 | up |
| MAF1 | 1.687690086 | 2.67E-12 | up |
| BAG6 | 1.687298295 | 3.97E-15 | up |
| UBXN6 | 1.686028701 | 2.12E-10 | up |
| GDE1 | 1.684563209 | 7.56E-29 | up |
| AQP10 | 1.679808435 | 8.53E-05 | up |
| MYL9 | 1.679450573 | 1.14E-06 | up |
| IFNGR1 | 1.678214658 | 1.27E-34 | up |
| DDB1 | 1.676213308 | 3.83E-17 | up |
| ITGA2B | 1.675051736 | 1.93E-07 | up |
| ENSG00000285635 | 1.674405255 | 8.95E-09 | up |
| MAP2K3 | 1.672361019 | 9.15E-19 | up |
| MFN2 | 1.671706709 | 4.16E-30 | up |
| WBP2 | 1.671466213 | 2.12E-17 | up |
| PLOD2 | 1.668927661 | 3.21E-06 | up |
| LHX4 | 1.668362502 | 0.00046749 | up |
| TPRG1L | 1.666171458 | 9.53E-19 | up |
| CXCL5 | 1.66180287 | 7.58E-06 | up |
| ELOF1 | 1.659550694 | 5.53E-12 | up |
| QPCT | 1.658581042 | 5.59E-20 | up |
| LTF | 1.655950718 | 1.57E-07 | up |
| RSPH14 | 1.65469407 | 2.47E-11 | up |
| ACSS3 | 1.650796648 | 2.45E-14 | up |
| ASPM | 1.650506875 | 1.06E-12 | up |
| AQP9 | 1.648830276 | 3.03E-26 | up |
| CYP1B1 | 1.647272084 | 6.47E-12 | up |
| NFKBIA | 1.646092096 | 3.67E-21 | up |
| SH3PXD2B | 1.645434641 | 2.57E-07 | up |
| SERPINB10 | 1.643942814 | 2.29E-05 | up |
| H2AJ | 1.641969502 | 1.72E-11 | up |
| RILP | 1.641410873 | 8.81E-11 | up |
| SLC31A2 | 1.640302777 | 1.85E-19 | up |
| BIRC2 | 1.637190516 | 2.34E-27 | up |
| NLRC4 | 1.634473525 | 1.69E-21 | up |
| HRH2 | 1.631073073 | 4.73E-18 | up |
| SLC22A4 | 1.630741824 | 5.69E-25 | up |
| GLUL | 1.628049494 | 1.84E-26 | up |
| LPL | 1.622442912 | 7.05E-05 | up |
| TENM1 | 1.620746842 | 3.21E-06 | up |
| KLHL8 | 1.619010305 | 5.11E-20 | up |
| CYSTM1 | 1.618381374 | 8.05E-15 | up |
| NLRP6 | 1.618167213 | 1.49E-13 | up |
| ALPL | 1.617452268 | 3.31E-13 | up |
| BCAT1 | 1.616626379 | 3.76E-12 | up |
| NECAB1 | 1.616317027 | 0.000580889 | up |
| WDR26 | 1.616266181 | 5.83E-39 | up |
| GATA1 | 1.616018923 | 1.18E-12 | up |
| CPD | 1.614523124 | 2.26E-18 | up |
| MCOLN1 | 1.613890916 | 1.79E-11 | up |
| GCLC | 1.613247201 | 3.06E-18 | up |
| CEACAM4 | 1.611621715 | 6.26E-13 | up |
| NPRL3 | 1.607190918 | 6.39E-11 | up |
| SLC11A1 | 1.604604316 | 9.37E-16 | up |
| FCGR1A | 1.602683518 | 8.30E-08 | up |
| VNN2 | 1.602181606 | 6.18E-18 | up |
| KIF14 | 1.600010381 | 1.97E-09 | up |
| KCNH2 | 1.597676537 | 1.02E-05 | up |
| NAMPT | 1.595759891 | 2.34E-26 | up |
| BCL6 | 1.594704621 | 5.13E-16 | up |
| CDH1 | 1.594600273 | 1.12E-07 | up |
| TREML1 | 1.592614599 | 6.23E-07 | up |
| MAPK14 | 1.59067447 | 2.30E-26 | up |
| MEGF9 | 1.586634978 | 3.72E-23 | up |
| KBTBD7 | 1.586025988 | 2.99E-16 | up |
| JDP2 | 1.585423961 | 7.80E-16 | up |
| ENSG00000285082 | 1.58513996 | 4.77E-14 | up |
| EIF4E3 | 1.584968922 | 7.55E-05 | up |
| TSC22D3 | 1.584598999 | 9.61E-22 | up |
| THRB | 1.58421643 | 4.07E-08 | up |
| MARCHF2 | 1.583463992 | 1.22E-15 | up |
| FEM1A | 1.581433419 | 6.22E-20 | up |
| ACP5 | 1.579152521 | 1.18E-07 | up |
| GYPE | 1.578994575 | 2.51E-10 | up |
| PHTF1 | 1.57896756 | 2.86E-18 | up |
| SAMD14 | 1.57715923 | 3.37E-05 | up |
| SLC25A39 | 1.574213056 | 3.68E-10 | up |
| MPIG6B | 1.573371743 | 3.31E-07 | up |
| ASCC2 | 1.572365089 | 2.10E-11 | up |
| ATG9A | 1.570484277 | 2.53E-18 | up |
| TLR8 | 1.56738337 | 1.24E-26 | up |
| PRKAR2B | 1.565315637 | 2.29E-12 | up |
| RMND5A | 1.564041188 | 1.45E-24 | up |
| HAGH | 1.563587725 | 9.10E-11 | up |
| ARHGAP24 | 1.562236242 | 1.37E-15 | up |
| GUK1 | 1.561399595 | 5.88E-10 | up |
| FOLR3 | 1.561140102 | 4.62E-06 | up |
| ETS2 | 1.560544589 | 5.89E-29 | up |
| FBXO7 | 1.559308096 | 8.90E-14 | up |
| LCN2 | 1.557956177 | 1.96E-07 | up |
| RNF123 | 1.557872114 | 5.74E-16 | up |
| S100A9 | 1.557196527 | 3.83E-16 | up |
| NFIL3 | 1.556463398 | 5.74E-27 | up |
| PDZK1IP1 | 1.553681813 | 1.26E-07 | up |
| UBE2B | 1.553263708 | 2.87E-39 | up |
| TRIM9 | 1.551962129 | 1.24E-09 | up |
| PRUNE2 | 1.551302986 | 4.18E-05 | up |
| JUND | 1.550607613 | 2.37E-15 | up |
| TMC5 | 1.549628897 | 3.20E-05 | up |
| LOXHD1 | 1.548660141 | 6.93E-10 | up |
| SH3GLB1 | 1.545431239 | 9.67E-37 | up |
| STK11 | 1.541504763 | 6.60E-14 | up |
| DYNLL1 | 1.540124827 | 5.10E-12 | up |
| RIPOR3 | 1.538785661 | 3.02E-06 | up |
| CACNA1E | 1.537377635 | 7.61E-14 | up |
| GHITM | 1.537186488 | 4.72E-21 | up |
| OOSP3 | 1.536172739 | 1.43E-09 | up |
| SPX | 1.535587833 | 1.54E-06 | up |
| H2BC18 | 1.53520188 | 3.28E-17 | up |
| SELP | 1.535043267 | 1.65E-16 | up |
| XPO7 | 1.533279171 | 1.66E-20 | up |
| RBM38 | 1.5305195 | 7.16E-09 | up |
| HAL | 1.529415482 | 1.09E-14 | up |
| SLC16A1 | 1.526963294 | 8.80E-10 | up |
| ITPKC | 1.525335752 | 9.49E-10 | up |
| SLC22A15 | 1.524843878 | 2.40E-21 | up |
| ST6GALNAC4 | 1.521479817 | 4.28E-08 | up |
| SLA | 1.521144298 | 1.60E-17 | up |
| LGALSL | 1.520465255 | 3.29E-15 | up |
| LYL1 | 1.515926079 | 1.09E-09 | up |
| FPR2 | 1.515561217 | 1.20E-22 | up |
| SMOX | 1.514592757 | 1.31E-09 | up |
| LRP10 | 1.514321675 | 2.34E-27 | up |
| IFT56 | 1.513930079 | 8.21E-07 | up |
| CMAS | 1.513867161 | 2.28E-16 | up |
| ENSG00000259753 | 1.513283118 | 4.85E-06 | up |
| TPX2 | 1.512878026 | 9.93E-10 | up |
| CMTM5 | 1.512856614 | 5.45E-07 | up |
| C9orf40 | 1.511873806 | 1.43E-11 | up |
| FAXDC2 | 1.510831028 | 1.22E-07 | up |
| OLR1 | 1.509364026 | 8.68E-05 | up |
| AIM2 | 1.50862317 | 1.75E-11 | up |
| RANBP10 | 1.505775835 | 9.55E-15 | up |
| GYG1 | 1.504836437 | 1.29E-13 | up |
| ENSG00000282988 | 1.504157022 | 5.11E-13 | up |
| SRRD | 1.503788281 | 2.46E-13 | up |
| FTH1 | 1.502891418 | 4.83E-14 | up |
| H2BC4 | 1.501597506 | 6.95E-28 | up |
| BTF3 | 1.501119171 | 3.29E-12 | up |
| ALOX15 | -3.27334016 | 6.28E-12 | down |
| PRSS33 | -2.973359843 | 5.94E-10 | down |
| SIGLEC8 | -2.889104757 | 4.86E-10 | down |
| SMPD3 | -2.70615125 | 2.75E-12 | down |
| PLAAT5 | -2.209571661 | 1.65E-10 | down |
| ZNF683 | -2.183173804 | 4.96E-10 | down |
| CLC | -2.183021621 | 1.13E-09 | down |
| PTGDR2 | -2.137382388 | 5.42E-09 | down |
| OLIG2 | -2.103358622 | 1.05E-06 | down |
| HRH4 | -1.929906736 | 5.31E-11 | down |
| TCF7 | -1.764162649 | 2.55E-30 | down |
| SPNS3 | -1.757662257 | 1.83E-07 | down |
| PLD4 | -1.735417411 | 1.14E-14 | down |
| NOG | -1.729518402 | 3.54E-07 | down |
| IL5RA | -1.728853832 | 8.81E-08 | down |
| LRRN3 | -1.651363311 | 1.04E-09 | down |
| CYSLTR2 | -1.552683541 | 2.95E-10 | down |
| TRABD2A | -1.503197322 | 1.77E-20 | down |

Table represents the differential gene expression analysis, including log_2_ fold changes and adjusted *P*-values. The log_2_FC represents the magnitude and direction of expression, with positive values indicating upregulation and negative values indicating downregulation. The adjusted *P*-values were computed using the Benjamini-Hochberg method to control the false discovery rate across multiple comparisons. Genes with *P*-adjusted < 0.05 were considered significantly differentially expressed.

**Table S3**. **The top 10 differentially expressed mRNAs between Mild HAPE patient’s vs controls**

| **Gene Upregulated** | **log_2_FC** | **Adjusted P-Value** | **Gene Downregulated** | **log_2_FC** | **Adjusted P-Value** |
| --- | --- | --- | --- | --- | --- |
| ARG1 | 3.864832543 | 5.83E-24 | PRSS33 | -4.4746196 | 1.23E-12 |
| CLEC4E | 3.047669727 | 6.67E-26 | SMPD3 | -3.433445233 | 2.95E-13 |
| FKBP5 | 3.140248662 | 2.18E-25 | ALOX15 | -4.086664894 | 4.85E-12 |
| SH3GLB1 | 1.682084948 | 5.18E-29 | NELL2 | -1.551233609 | 1.30E-12 |
| TLR2 | 2.731811545 | 3.56E-36 | TCF7 | -1.747548026 | 2.27E-19 |
| IL13RA1 | 1.82589099 | 4.74E-29 | TRABD2A | -1.579790226 | 9.30E-15 |
| IFNGR1 | 1.816575139 | 7.02E-27 | ST8SIA1 | -1.579006065 | 1.47E-12 |
| FOXO3 | 1.638378265 | 2.18E-25 | WNT7A | -1.844537072 | 1.27E-14 |
| METTL9 | 1.804346415 | 8.80E-24 | BICDL1 | -1.500064442 | 3.12E-20 |
| MAP1LC3B | 1.735912268 | 8.80E-24 | MAN1C1 | -1.530416633 | 1.87E-17 |

The log_2_ fold changes represents the magnitude and direction of expression, with positive values indicating upregulation and negative values indicating downregulation. The adjusted *P-* values were computed using the Benjamini-Hochberg method to control the false discovery rate across multiple comparisons. Genes with *P*-adjusted < 0.05 were considered significantly differentially expressed.

**Table S4. The top 10 differentially expressed mRNAs between Moderate HAPE patient’s vs controls**

| **Gene Upregulated** | **log_2_FC** | **Adjusted P-Value** | **Gene Downregulated** | **log_2_FC** | **Adjusted P-Value** |
| --- | --- | --- | --- | --- | --- |
| METTL9 | 2.368394453 | 3.79E-35 | NELL2 | -1.517252203 | 1.68E-10 |
| HIPK1 | 1.571417784 | 5.38E-33 | CD28 | -1.69619875 | 5.83E-18 |
| UBE2H | 2.217273772 | 5.89E-36 | TCF7 | -1.800327854 | 8.04E-18 |
| STOM | 2.694493224 | 1.30E-32 | TRABD2A | -1.514344536 | 8.69E-12 |
| SNX3 | 2.337048644 | 1.29E-30 | MYBL1 | -1.681731128 | 7.61E-10 |
| FOXO3 | 2.202175506 | 3.61E-39 | IL21R | -1.515195444 | 3.03E-18 |
| WDR26 | 1.860490395 | 1.30E-32 | SBK1 | -1.657049164 | 1.09E-10 |
| TCP11L2 | 1.751728994 | 1.14E-30 | ZNF239 | -1.718711313 | 8.57E-09 |
| MAP1LC3B | 2.212062183 | 4.04E-33 | P2RY10 | -1.570946059 | 1.30E-14 |
| CCNDBP1 | 2.436713838 | 9.50E-32 | LRRN3 | -2.0556852 | 2.54E-08 |

The log_2_ fold changes represents the magnitude and direction of expression, with positive values indicating upregulation and negative values indicating downregulation. The adjusted *P-* values were computed using the Benjamini-Hochberg method to control the false discovery rate across multiple comparisons. Genes with *P*-adjusted < 0.05 were considered significantly differentially expressed.

**Table S5. The top 10 differentially expressed mRNAs between Severe HAPE patient’s vs controls**

| **Gene Upregulated** | **log_2_FC** | **Adjusted P-Value** | **Gene Downregulated** | **log_2_FC** | **Adjusted P-Value** |
| --- | --- | --- | --- | --- | --- |
| TLR2 | 2.420286868 | 5.28E-30 | TCF7 | -1.756489012 | 1.45E-20 |
| ARG1 | 4.443439415 | 6.02E-33 | CLC | -2.231643487 | 8.24E-08 |
| IRAK3 | 2.794971928 | 2.95E-29 | IL5RA | -2.091788237 | 3.93E-08 |
| STOM | 2.259859151 | 3.70E-28 | SMPD3 | -2.760740109 | 1.36E-09 |
| UBE2H | 1.753969579 | 6.35E-28 | PTGDR2 | -2.370839688 | 1.89E-07 |
| WDR26 | 1.63530484 | 1.32E-30 | CYSLTR2 | -1.669380934 | 3.00E-08 |
| CCNDBP1 | 2.099043948 | 7.61E-29 | HRH4 | -1.950908353 | 4.63E-08 |
| CPEB4 | 1.521505599 | 2.74E-29 | PLD4 | -1.602719725 | 1.11E-08 |
| FOXO3 | 1.821459345 | 6.87E-33 | ALOX15 | -3.596949992 | 2.20E-10 |
| UBE2B | 1.559086839 | 9.90E-29 | PRSS33 | -2.971312904 | 1.07E-07 |

The log_2_ fold changes represents the magnitude and direction of expression, with positive values indicating upregulation and negative values indicating downregulation. The adjusted *P-* values were computed using the Benjamini-Hochberg method to control the false discovery rate across multiple comparisons. Genes with *P*-adjusted < 0.05 were considered significantly differentially expressed.

**Table S6.** **The top 10 hub genes derived from the eleven algorithms of cytohubba between HAPE patient’s vs controls**

| **Cytohubba algorithms** | **Genes** | **log2FC** | **Adjusted**  **P-Value** |
| --- | --- | --- | --- |
| MCC | EPB42 | 2.598122913 | 2.12E-18 |
|  | SPTA1 | 2.738075355 | 1.49E-22 |
|  | ALAS2 | 3.103795425 | 1.47E-22 |
|  | GYPA | 3.49533689 | 9.40E-18 |
|  | GATA1 | 1.616018923 | 1.18E-12 |
|  | SLC4A1 | 2.406722669 | 1.39E-17 |
|  | RHAG | 2.608932125 | 7.81E-12 |
|  | AHSP | 3.72676422 | 2.75E-24 |
|  | GYPB | 2.841071065 | 7.71E-17 |
|  | KLF1 | 1.740932832 | 4.48E-08 |
| DMNC | RHD | 2.351620907 | 7.76E-14 |
|  | HEMGN | 2.235060734 | 1.08E-15 |
|  | SPTA1 | 2.738075355 | 1.49E-22 |
|  | TSPO2 | 2.378051711 | 8.02E-09 |
|  | GYPE | 1.578994575 | 2.51E-10 |
|  | TRIM10 | 2.234294757 | 3.02E-14 |
|  | TMCC2 | 3.274023791 | 3.73E-30 |
|  | ACKR1 | 2.113961484 | 2.63E-10 |
|  | HBD | 4.070297139 | 2.14E-26 |
|  | KEL | 2.050358554 | 1.66E-13 |
| MNC | EPB42 | 2.598122913 | 2.12E-18 |
|  | TLR2 | 2.517274855 | 6.81E-45 |
|  | MMP9 | 2.003084924 | 2.52E-11 |
|  | TFRC | 1.731907498 | 3.80E-13 |
|  | GATA1 | 1.616018923 | 1.18E-12 |
|  | SLC4A1 | 2.406722669 | 1.39E-17 |
|  | SNCA | 1.906456804 | 2.23E-14 |
|  | FECH | 2.527447972 | 2.67E-20 |
|  | GYPB | 2.841071065 | 7.71E-17 |
|  | KLF1 | 1.740932832 | 4.48E-08 |
| Degree | EPB42 | 2.598122913 | 2.12E-18 |
|  | TLR2 | 2.517274855 | 6.81E-45 |
|  | ALAS2 | 3.103795425 | 1.47E-22 |
|  | MMP9 | 2.003084924 | 2.52E-11 |
|  | TFRC | 1.731907498 | 3.80E-13 |
|  | GATA1 | 1.616018923 | 1.18E-12 |
|  | SLC4A1 | 2.406722669 | 1.39E-17 |
|  | SNCA | 1.906456804 | 2.23E-14 |
|  | FECH | 2.527447972 | 2.67E-20 |
|  | GYPB | 2.841071065 | 7.71E-17 |
| EPC | EPB42 | 2.598122913 | 2.12E-18 |
|  | ALAS2 | 3.103795425 | 1.47E-22 |
|  | GYPA | 3.49533689 | 9.40E-18 |
|  | GATA1 | 1.616018923 | 1.18E-12 |
|  | SLC4A1 | 2.406722669 | 1.39E-17 |
|  | AHSP | 3.72676422 | 2.75E-24 |
|  | SNCA | 1.906456804 | 2.23E-14 |
|  | FECH | 2.527447972 | 2.67E-20 |
|  | GYPB | 2.841071065 | 7.71E-17 |
|  | KLF1 | 1.740932832 | 4.48E-08 |
| Bottleneck | LTF | 1.655950718 | 1.57E-07 |
|  | BCL2L1 | 2.074891415 | 1.17E-18 |
|  | TFRC | 1.731907498 | 3.80E-13 |
|  | GATA1 | 1.616018923 | 1.18E-12 |
|  | ZBTB16 | 2.440131187 | 9.82E-14 |
|  | ITGA2B | 1.675051736 | 1.93E-07 |
|  | ABCG2 | 2.11899388 | 3.71E-14 |
|  | UBB | 2.057976683 | 4.70E-12 |
|  | SNCA | 1.906456804 | 2.23E-14 |
|  | FECH | 2.527447972 | 2.67E-20 |
| Eccentricity | NFKBIA | 1.646092096 | 3.67E-21 |
|  | IL1R2 | 3.214205267 | 6.03E-33 |
|  | MAP2K3 | 1.672361019 | 9.15E-19 |
|  | CDH1 | 1.594600273 | 1.12E-07 |
|  | TLR8 | 1.56738337 | 1.24E-26 |
|  | JUND | 1.550607613 | 2.37E-15 |
|  | CEBPD | 1.761657661 | 1.43E-17 |
|  | CXCL5 | 1.66180287 | 7.58E-06 |
|  | BCL6 | 1.594704621 | 5.13E-16 |
|  | TSC22D3 | 1.584598999 | 9.61E-22 |
| Closeness | BCL2L1 | 2.074891415 | 1.17E-18 |
|  | GYPA | 3.49533689 | 9.40E-18 |
|  | GATA1 | 1.616018923 | 1.18E-12 |
|  | MMP9 | 2.003084924 | 2.52E-11 |
|  | TFRC | 1.731907498 | 3.80E-13 |
|  | FOXO3 | 1.868356509 | 1.06E-42 |
|  | SLC4A1 | 2.406722669 | 1.39E-17 |
|  | SNCA | 1.906456804 | 2.23E-14 |
|  | FECH | 2.527447972 | 2.67E-20 |
|  | KLF1 | 1.740932832 | 4.48E-08 |
| Radiality | BCL2L1 | 2.074891415 | 1.17E-18 |
|  | GYPA | 3.49533689 | 9.40E-18 |
|  | GATA1 | 1.616018923 | 1.18E-12 |
|  | MMP9 | 2.003084924 | 2.52E-11 |
|  | TFRC | 1.731907498 | 3.80E-13 |
|  | FOXO3 | 1.868356509 | 1.06E-42 |
|  | SLC4A1 | 2.406722669 | 1.39E-17 |
|  | SNCA | 1.906456804 | 2.23E-14 |
|  | FECH | 2.527447972 | 2.67E-20 |
|  | KLF1 | 1.740932832 | 4.48E-08 |
| Betweenness | TLR2 | 2.517274855 | 6.81E-45 |
|  | BCL2L1 | 2.074891415 | 1.17E-18 |
|  | MMP9 | 2.003084924 | 2.52E-11 |
|  | GATA1 | 1.616018923 | 1.18E-12 |
|  | TFRC | 1.731907498 | 3.80E-13 |
|  | FOXO3 | 1.868356509 | 1.06E-42 |
|  | ITGA2B | 1.675051736 | 1.93E-07 |
|  | UBB | 2.057976683 | 4.70E-12 |
|  | SNCA | 1.906456804 | 2.23E-14 |
|  | FECH | 2.527447972 | 2.67E-20 |
| Stress | TLR2 | 2.517274855 | 6.81E-45 |
|  | BCL2L1 | 2.074891415 | 1.17E-18 |
|  | MMP9 | 2.003084924 | 2.52E-11 |
|  | GATA1 | 1.616018923 | 1.18E-12 |
|  | TFRC | 1.731907498 | 3.80E-13 |
|  | FOXO3 | 1.868356509 | 1.06E-42 |
|  | ITGA2B | 1.675051736 | 1.93E-07 |
|  | UBB | 2.057976683 | 4.70E-12 |
|  | SNCA | 1.906456804 | 2.23E-14 |
|  | FECH | 2.527447972 | 2.67E-20 |

Table represents the top 10 hub genes present in each algorithm of cytohubba plug in of cytoscape. The log_2_ fold changes represents the magnitude and direction of expression, with positive values indicating upregulation and negative values indicating downregulation. The adjusted *P*-values were computed using the Benjamini-Hochberg method to control the false discovery rate across multiple comparisons. Genes with *P*-adjusted < 0.05 were considered significantly differentially expressed.

**Table S7. The ROC analysis of hub genes derived from the PPI network between HAPE patient’s vs controls**

| **Genes** | **P -Value** | **AUC**  **(95% CI)** | **Sensitivity (%)** | **Specificity (%)** |
| --- | --- | --- | --- | --- |
| TLR2 | <0.0001 | 0.9962 | 100 | 100 |
| FOXO3 | <0.0001 | 0.9937 | 100 | 100 |
| ALAS2 | <0.0001 | 0.9687 | 100 | 100 |
| SPTA1 | <0.0001 | 0.9662 | 100 | 100 |
| SLC4A1 | <0.0001 | 0.9524 | 97.62 | 94.74 |
| MMP9 | <0.0001 | 0.9474 | 97.62 | 94.74 |
| BCL2L1 | <0.0001 | 0.9436 | 97.62 | 94.74 |
| AHSP | <0.0001 | 0.9424 | 97.62 | 94.74 |
| EPB42 | <0.0001 | 0.9373 | 95.24 | 94.74 |
| FECH | <0.0001 | 0.9336 | 95.24 | 94.74 |
| SNCA | <0.0001 | 0.9185 | 95.24 | 89.47 |
| GYPB | <0.0001 | 0.9035 | 92.86 | 89.47 |
| GATA1 | <0.0001 | 0.8985 | 92.86 | 89.47 |
| UBB | <0.0001 | 0.8947 | 92.86 | 89.47 |
| GYPA | <0.0001 | 0.8915 | 92.86 | 88.89 |
| TFRC | <0.0001 | 0.8759 | 90.48 | 89.47 |
| ITGA2B | <0.0001 | 0.8283 | 85.71 | 84.21 |
| KLF1 | <0.0001 | 0.8183 | 85.71 | 84.21 |

Table represent the ROC of hub genes derived from PPI network. The Area under curve measures the gene’s ability to distinguish between disease and control groups, with values closer to 1 indicating stronger discrimination. Sensitivity represents the true positive rate, while specificity indicates the true negative rate. *P*-values < 0.05 were considered significant.

**Table S8. Differentially expressed lncRNAs between HAPE patient’s vs controls**

| **Genes** | **log2FC** | **Adjusted**  **P-Value** | **Expression type** |
| --- | --- | --- | --- |
| ENSG00000288853 | 4.311840906 | 2.54E-29 | up |
| ENSG00000267780 | 3.705473767 | 2.31E-11 | up |
| LINC02207 | 3.388515299 | 3.72E-25 | up |
| LINC01093 | 3.285945213 | 3.12E-13 | up |
| ENSG00000254420 | 3.107628161 | 6.17E-32 | up |
| ENSG00000287255 | 3.069254564 | 2.20E-26 | up |
| ENSG00000268119 | 3.064543732 | 2.16E-17 | up |
| ENSG00000289456 | 3.031030106 | 4.01E-12 | up |
| LINC01580 | 3.030308402 | 9.29E-11 | up |
| ENSG00000249309 | 3.016852597 | 2.87E-42 | up |
| ENSG00000287216 | 3.012811453 | 4.51E-30 | up |
| ENSG00000285534 | 3.00575818 | 7.17E-14 | up |
| ENSG00000255801 | 2.974475987 | 2.50E-28 | up |
| ENSG00000286342 | 2.941123312 | 2.31E-11 | up |
| ENSG00000285117 | 2.903695477 | 4.27E-22 | up |
| ENSG00000289242 | 2.894304995 | 4.62E-28 | up |
| ENSG00000287642 | 2.84013285 | 1.18E-15 | up |
| ENSG00000229160 | 2.830743499 | 3.85E-11 | up |
| ENSG00000290003 | 2.817090596 | 3.67E-17 | up |
| CLRN1-AS1 | 2.806694946 | 6.20E-10 | up |
| ENSG00000288836 | 2.795708756 | 1.90E-12 | up |
| LINC02967 | 2.773594373 | 1.23E-09 | up |
| ENSG00000289514 | 2.581261144 | 2.39E-13 | up |
| ENSG00000287771 | 2.553573946 | 3.04E-24 | up |
| LINC01127 | 2.537609843 | 6.86E-21 | up |
| ENSG00000282840 | 2.529140118 | 2.98E-11 | up |
| XIST | 2.507596882 | NA | ns |
| ENSG00000289066 | 2.500190925 | 4.10E-15 | up |
| ENSG00000268170 | 2.486969914 | 1.08E-17 | up |
| ESRG | 2.45701685 | 7.39E-06 | up |
| ENSG00000286076 | 2.45147806 | 4.51E-30 | up |
| ENSG00000288887 | 2.449496763 | 2.70E-34 | up |
| ENSG00000250906 | 2.445694499 | 1.62E-12 | up |
| LINC02772 | 2.438146269 | 4.86E-12 | up |
| ENSG00000289347 | 2.413651346 | 7.51E-16 | up |
| ENSG00000289933 | 2.354890524 | 5.19E-06 | up |
| ENSG00000285966 | 2.345578238 | 1.67E-12 | up |
| ENSG00000228352 | 2.32024697 | 4.38E-13 | up |
| ENSG00000268938 | 2.300110151 | 4.46E-12 | up |
| ENSG00000285984 | 2.29409141 | 9.64E-11 | up |
| ENSG00000265401 | 2.262013317 | 7.77E-08 | up |
| PHC2-AS1 | 2.240325182 | 4.30E-13 | up |
| LINC02940 | 2.237687919 | 3.61E-09 | up |
| ENSG00000229308 | 2.236619701 | 3.53E-07 | up |
| ENSG00000258473 | 2.227265872 | 1.38E-15 | up |
| ABALON | 2.224603782 | 6.06E-10 | up |
| ENSG00000287317 | 2.213964342 | 3.44E-06 | up |
| KDM7A-DT | 2.206388508 | 2.38E-15 | up |
| ENSG00000251139 | 2.196767893 | 1.09E-13 | up |
| BASP1-AS1 | 2.184181793 | 2.07E-20 | up |
| ENSG00000289197 | 2.178150258 | 6.40E-13 | up |
| ENSG00000290441 | 2.177585172 | 4.54E-16 | up |
| ENSG00000167046 | 2.164452783 | 1.26E-06 | up |
| NUDT16-DT | 2.126262521 | 1.21E-20 | up |
| ENSG00000257258 | 2.125667024 | 3.12E-26 | up |
| LINC02972 | 2.108588283 | 2.39E-09 | up |
| FCGR1BP | 2.0977645 | 4.30E-15 | up |
| ENSG00000259268 | 2.078863463 | 5.90E-10 | up |
| ENSG00000287860 | 2.075164024 | 4.03E-10 | up |
| ENSG00000287670 | 2.03628518 | 4.49E-09 | up |
| NUDT16L2P | 2.035667145 | 1.03E-13 | up |
| ENSG00000289298 | 2.026268444 | 6.39E-06 | up |
| ENSG00000289525 | 2.012949154 | 2.88E-20 | up |
| ENSG00000248774 | 2.001680914 | 6.34E-21 | up |
| ENSG00000290010 | 1.999259051 | 2.46E-10 | up |
| KCNJ2-AS1 | 1.99413557 | 3.79E-19 | up |
| ENSG00000290556 | 1.992980585 | 1.19E-12 | up |
| ENSG00000286256 | 1.991126353 | 3.22E-13 | up |
| ENSG00000253214 | 1.978380763 | 7.45E-10 | up |
| SNCA-AS1 | 1.973259506 | 9.03E-06 | up |
| C3orf86P | 1.960554089 | 1.76E-17 | up |
| MIR3945HG | 1.960110365 | 4.40E-23 | up |
| ENSG00000270972 | 1.950364357 | 0.00132186 | up |
| ENSG00000288025 | 1.917686639 | 3.65E-12 | up |
| ENSG00000256357 | 1.913007849 | 2.91E-24 | up |
| ENSG00000254468 | 1.909382363 | 1.06E-09 | up |
| MYO16-AS1 | 1.904748386 | 1.17E-05 | up |
| CA3-AS1 | 1.892415234 | 8.34E-08 | up |
| ENSG00000243797 | 1.88462861 | 7.61E-14 | up |
| ENSG00000230492 | 1.882565172 | 2.96E-11 | up |
| CYP1B1-AS1 | 1.863498971 | 2.28E-19 | up |
| ENSG00000288762 | 1.850324481 | 2.75E-06 | up |
| ENSG00000287360 | 1.842972204 | 0.003867412 | up |
| LINC02555 | 1.838733368 | 1.33E-05 | up |
| ENSG00000288819 | 1.83757733 | 1.67E-08 | up |
| ENSG00000268833 | 1.834963748 | 2.09E-14 | up |
| ENSG00000255328 | 1.822807627 | 5.59E-05 | up |
| ENSG00000289150 | 1.817526802 | 4.96E-08 | up |
| ENSG00000225313 | 1.816466872 | 1.21E-11 | up |
| LINC00937 | 1.800708925 | 1.98E-13 | up |
| ENSG00000293500 | 1.793931436 | 3.38E-11 | up |
| ENSG00000284930 | 1.786520548 | 3.40E-12 | up |
| ENSG00000267062 | 1.7857192 | 5.34E-06 | up |
| ENSG00000275527 | 1.752210281 | 2.09E-06 | up |
| ENSG00000275741 | 1.748785165 | 4.57E-13 | up |
| LINC01220 | 1.74565234 | 3.42E-11 | up |
| ENSG00000265907 | 1.742035731 | 1.04E-07 | up |
| ENSG00000254695 | 1.741184021 | 2.65E-08 | up |
| ENSG00000287632 | 1.740090921 | 6.91E-06 | up |
| ENSG00000289573 | 1.738455395 | 3.08E-06 | up |
| ENSG00000257715 | 1.732577549 | 2.59E-08 | up |
| ENSG00000254789 | 1.709881941 | 1.80E-07 | up |
| ENSG00000257878 | 1.706489829 | 5.45E-15 | up |
| ENSG00000231039 | 1.698419771 | 7.33E-06 | up |
| RUNDC3A-AS1 | 1.690402224 | 2.93E-05 | up |
| LINC00570 | 1.68234445 | 1.37E-08 | up |
| ENSG00000236911 | 1.67434986 | 1.61E-09 | up |
| CCR5AS | 1.671371662 | 2.09E-07 | up |
| LINC02217 | 1.666373875 | 1.45E-06 | up |
| ENSG00000291221 | 1.658972701 | 6.10E-07 | up |
| FLJ46284 | 1.658227182 | 4.41E-14 | up |
| ENSG00000289351 | 1.654422017 | 7.11E-17 | up |
| ENSG00000268555 | 1.65387939 | 4.40E-07 | up |
| ENSG00000238009 | 1.650730688 | 6.10E-10 | up |
| ENSG00000237525 | 1.642875528 | 0.004707003 | up |
| WDFY3-AS2 | 1.634332853 | 1.83E-13 | up |
| VNN3P | 1.633236539 | 2.11E-11 | up |
| ENSG00000290421 | 1.628286903 | 7.54E-06 | up |
| ENSG00000268240 | 1.620411003 | 3.89E-05 | up |
| ENSG00000280136 | 1.616580464 | 4.50E-10 | up |
| ENSG00000259182 | 1.6152823 | 2.11E-07 | up |
| NFE4 | 1.614497972 | 3.08E-15 | up |
| ENSG00000285557 | 1.611313304 | 4.52E-08 | up |
| ENSG00000282024 | 1.607695555 | 8.17E-05 | up |
| ENSG00000288954 | 1.607215308 | 2.20E-15 | up |
| LINC02288 | 1.600521816 | 1.72E-15 | up |
| ENSG00000251393 | 1.597483754 | 7.21E-11 | up |
| LINC00862 | 1.586848326 | 3.33E-17 | up |
| ENSG00000288932 | 1.58571514 | 3.63E-21 | up |
| ENSG00000290074 | 1.585270755 | 6.45E-10 | up |
| ENSG00000235840 | 1.581274624 | 2.00E-05 | up |
| DNAJC3-DT | 1.579410203 | 2.11E-13 | up |
| LINC02471 | 1.578838595 | 6.26E-05 | up |
| LINC02193 | 1.567934959 | 5.02E-07 | up |
| ENSG00000253736 | 1.562107281 | 4.79E-13 | up |
| LINC02751 | 1.561807651 | 4.24E-07 | up |
| ENSG00000225889 | 1.560924904 | 7.99E-15 | up |
| ENSG00000289091 | 1.559293645 | 1.23E-06 | up |
| ENSG00000289339 | 1.558456444 | 2.40E-05 | up |
| ENSG00000289541 | 1.552356803 | 8.61E-23 | up |
| ENSG00000287950 | 1.54913868 | 2.05E-07 | up |
| ENSG00000269191 | 1.548010694 | 0.000680329 | up |
| XPC-AS1 | 1.542684478 | 7.85E-17 | up |
| ENSG00000251095 | 1.541539262 | 4.65E-05 | up |
| ENSG00000258082 | 1.540352303 | 2.23E-10 | up |
| ENSG00000289382 | 1.539328273 | 1.80E-10 | up |
| ENSG00000288997 | 1.535810803 | 1.08E-05 | up |
| ENSG00000260592 | 1.533633744 | 3.42E-07 | up |
| ENSG00000290038 | 1.532894102 | 4.86E-07 | up |
| A2ML1-AS1 | 1.531801174 | 0.000389771 | up |
| ENSG00000251259 | 1.530290362 | 4.30E-15 | up |
| LINC00513 | 1.529510619 | 5.64E-11 | up |
| NAMPT-AS1 | 1.522644225 | 5.29E-07 | up |
| LNCATV | 1.519119463 | 3.50E-15 | up |
| ENSG00000285898 | 1.510619374 | 0.012489377 | up |
| ENSG00000293388 | 1.509853175 | 2.39E-09 | up |
| LINC03058 | 1.507631874 | 0.000299153 | up |
| ENSG00000290062 | 1.504363846 | 2.92E-09 | up |
| ENSG00000287644 | 1.502636608 | 1.31E-10 | up |
| ENSG00000289561 | -2.861107132 | 1.32E-06 | down |
| ENSG00000290017 | -2.705659561 | 5.66E-13 | down |
| ENSG00000250696 | -2.405193099 | 3.53E-06 | down |
| ENSG00000290427 | -2.247019886 | 4.50E-10 | down |
| RHOXF1P1 | -1.871201509 | 7.22E-06 | down |
| ENSG00000226423 | -1.754446628 | 4.19E-07 | down |
| C12orf75-AS1 | -1.740277686 | 1.60E-06 | down |
| ENSG00000289299 | -1.614368616 | 3.21E-07 | down |
| CCDC86-AS1 | -1.597172149 | 8.77E-06 | down |
| ENSG00000277301 | -1.561609463 | 3.89E-09 | down |
| UICLM | -1.546357054 | 4.11E-06 | down |
| LINC00402 | -1.531439203 | 4.25E-15 | down |
| ENSG00000225885 | -1.523607151 | 1.28E-09 | down |

Table represents the differential lncRNA analysis, including log_2_ fold changes and adjusted *P-*values. The log_2_FC represents the magnitude and direction of expression, with positive values indicating upregulation and negative values indicating downregulation. The adjusted *P*-values were computed using the Benjamini-Hochberg method to control the false discovery rate across multiple comparisons. Genes with *P*-adjusted < 0.05 were considered significantly differentially expressed.

**Table S9. WGCNA derived hub lncRNAs predicted function and disease associations**

| **Genes** | **Predicted function** | **Disease association** | **References** |
| --- | --- | --- | --- |
| LINC01093 | Regulate transcription and cell proliferation | Hepatocellular carcinoma: implicated in tumor progression and poor prognosis | [31] |
| LINC02863 | Regulate renal development by modulating PAX8–Wnt/β-catenin signaling | Antenatal hydronephrosis: LINC02863 levels are upregulated in patients | [32] |
| LINC02853 | Act in a cisplatin-resistance–related ceRNA regulatory network influencing mitochondrial-regulated apoptosis and immune (T-cell) infiltration | Small cell lung cancer: acts as a cisplatin resistance–related lncRNA | [33] |
| CFLAR-AS1 | Modulate immune response and apoptotic pathways by regulating CFLAR | Cancer and autoimmune disease: associated with immune dysregulation | [34] |
| LINC02128 | Implicated in pathways affecting immune infiltration and drug response. | Esophageal cancer: involved in anoikis resistance and tumor cell survival | [35] |
| TCL6 | Acts as a tumor suppressor, inhibits proliferation, modulates signaling pathways like EGFR/AKT and immune regulation | Renal cell carcinoma, breast cancer, and kidney cancer: low expression correlates with tumor progression and poor prognosis | [36-38] |
| UBOX5-AS1 | Promoting epithelial-mesenchymal transition (EMT), cell migration, and invasion by interacting with (HIF-1α) signaling pathways | Ovarian endometriosis: linked to aberrant cell growth and inflammation in ovarian tissue | [39] |
| FKBP14-AS1 | Regulate extracellular matrix stability and collagen processing | Ehlers-Danlos Syndrome: regulate FKBP14 expression which might impact connective tissue integrity | [40] |
| CDC42EP3-AS1 | Regulate cytoskeletal organization and cell migration through interaction with CDC42EP3 | Colorectal cancer: linked to invasion, metastasis, and cell motility | [41] |
| RHOXF1-AS1 | Modulate transcription factors related to RHOXF1 | Lung adenocarcinoma: serve as a prognostic biomarker in LUAD | [42] |
